# Growth form evolution and hybridization in *Senecio* (Asteraceae) from the high equatorial Andes

**DOI:** 10.1002/ece3.3206

**Published:** 2017-07-10

**Authors:** Eva Dušková, Petr Sklenář, Filip Kolář, Diana L. A. Vásquez, Katya Romoleroux, Tomáš Fér, Karol Marhold

**Affiliations:** ^1^ Department of Botany Faculty of Science Charles University Prague Czech Republic; ^2^ National Centre for Biosystematics Natural History Museum University of Oslo Oslo Norway; ^3^ Escuela de Ciencias Biológicas Pontificia Universidad Católica del Ecuador Quito Ecuador; ^4^ Institute of Botany Slovak Academy of Sciences Bratislava Slovak Republic

**Keywords:** adaptive radiation, Andes, *Culcitium*, growth forms, hybridization, *Lasiocephalus*, Neotropical montane forest, páramo, *Senecio*

## Abstract

Changes in growth forms frequently accompany plant adaptive radiations, including páramo–a high‐elevation treeless habitat type of the northern Andes. We tested whether diverse group of *Senecio* inhabiting montane forests and páramo represented such growth form changes. We also investigated the role of Andean geography and environment in structuring genetic variation of this group. We sampled 108 populations and 28 species of *Senecio* (focusing on species from former genera *Lasiocephalus* and *Culcitium*) and analyzed their genetic relationships and patterns of intraspecific variation using DNA fingerprinting (AFLPs) and nuclear DNA sequences (ITS). We partitioned genetic variation into environmental and geographical components. ITS‐based phylogeny supported monophyly of a *Lasiocephalus*‐*Culcitium* clade. A grade of herbaceous alpine *Senecio* species subtended the *Lasiocephalus*‐*Culcitium* clade suggesting a change from the herbaceous to the woody growth form. Both ITS sequences and the AFLPs separated a group composed of the majority of páramo subshrubs from other group(s) comprising both forest and páramo species of various growth forms. These morphologically variable group(s) further split into clades encompassing both the páramo subshrubs and forest lianas, indicating independent switches among the growth forms and habitats. The finest AFLP genetic structure corresponded to morphologically delimited species except in two independent cases in which patterns of genetic variation instead reflected geography. Several morphologically variable species were genetically admixed, which suggests possible hybrid origins. Latitude and longitude accounted for 5%–8% of genetic variation in each of three AFLP groups, while the proportion of variation attributed to environment varied between 8% and 31% among them. A change from the herbaceous to the woody growth form is suggested for species of high‐elevation Andean *Senecio*. Independent switches between habitats and growth forms likely occurred within the group. Hybridization likely played an important role in species diversification.

## 1 INTRODUCTION

The uplift of Andean cordilleras played a major role in promoting diversification of the Neotropical flora (Antonelli, Nylander, Persson, & Sanmartin, 2009; Hoorn et al., 2010; Hughes, Pennington, & Antonelli, 2013). The equatorial Andes in particular host a very diverse flora spanning a great variety of habitats between the montane forest and the alpine páramo belts (Churchill, Balslev, Forero, & Luteyn, 1995; Luteyn, 1999; Myers, Mittermaier, Mittermaier, da Fonseca, & Kent, 2000). Páramo habitats became available by the end of the north‐Andean orogeny ca. 3–5 Ma (van der Hammen & Cleef, 1986). In spite of its relative youth, the páramo flora is especially rich in groups which underwent radiations (Madriñán, Cortés, & Richardson, 2013; Sklenář, Dušková, & Balslev, 2011). Elevational shifts of vegetation belts during the Pleistocene, which repeatedly fragmented and reunited plant populations, were coupled with the final uplift of the Andes, which created new ecological opportunities and promoted diversification of the flora (Hughes & Eastwood, 2006; Jabaily & Sytsma, 2012; Luebert & Weigend, 2014; Madriñán et al., 2013).

Adaptation to newly emerging supra‐forest habitats has been an important source of functional diversity in the páramo flora (Sklenář et al., 2011). DNA‐based phylogenetic studies indicate that the north‐Andean genus *Espeletia* Mutis ex Bonpl. (Asteraceae) evolved distinct growth forms upon colonizing the páramo (Cuatrecasas, 1986; Panero, Jansen, & Clevinger, 1999; Rauscher, 2002). In *Aragoa* Kunth (Plantaginaceae), another genus endemic to the northern Andes, arborescent plants of the montane forest evolved into páramo shrubs (Bello, Chase, Olmstead, Rønsted, & Albach, 2002; Fernández‐Alonso, 1995), and growth form changes have been found in other Andean genera such as *Lupinus* L., *Hinterhubera* Sch. Bip. ex Wedd., *Laestadia* Kunth ex Less., and *Westoniella* Cuatrec. (Hughes & Eastwood, 2006; Karaman‐Castro & Urbatsch, 2009). However, deep insights into growth form evolution among north‐Andean plant groups, based upon genetic markers with sufficiently detailed resolution have been rare (Jabaily & Sytsma, 2012; Uribe‐Convers, Settles, & Tank, 2016).

**Figure 1:**
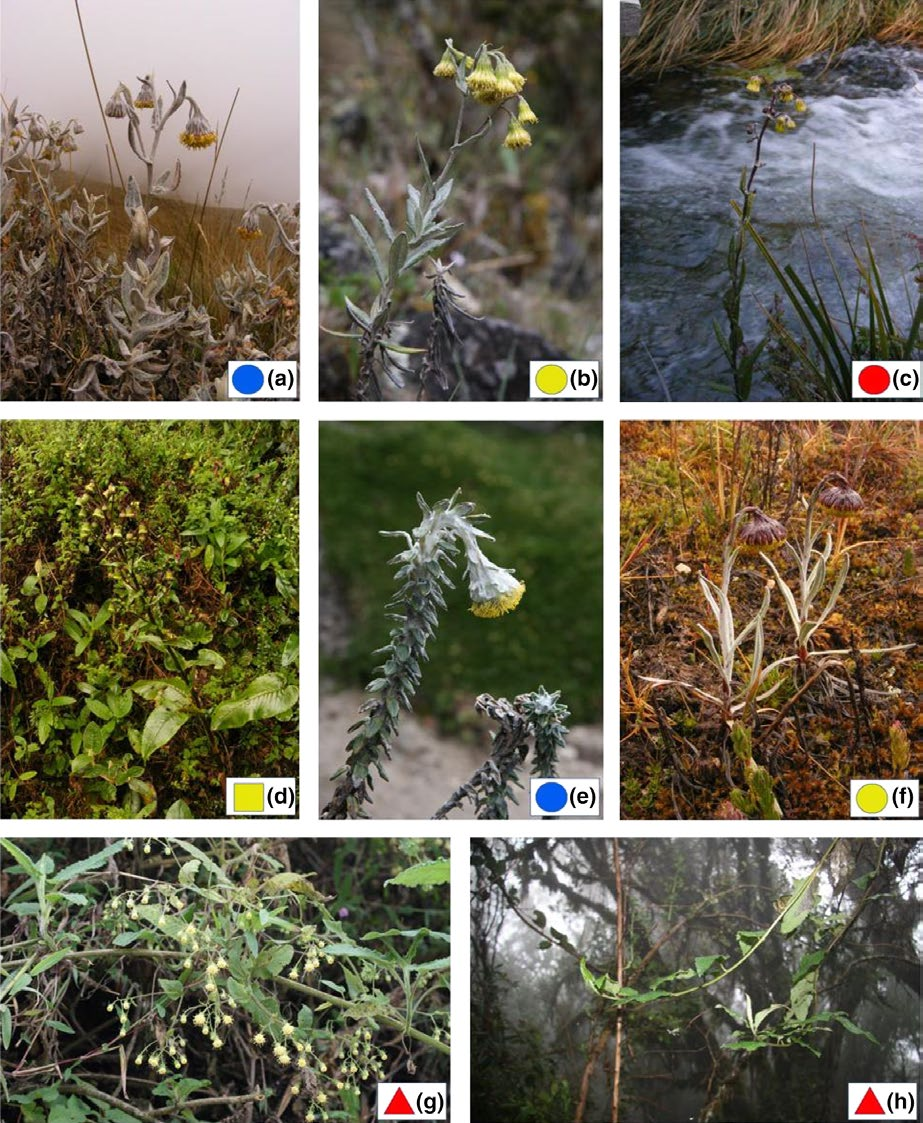
Growth form variation among the investigated *Senecio* species from the high Andes: (a) *S. lingulatus*, Ecuador, páramo; (b) *S. longepenicillatus,* Venezuela, superpáramo; (c) high‐elevation form of *S. otophorus,* Colombia, superpáramo; (d) *S. mojandensis*, Ecuador, páramo; (e) *S. superandinus*, Ecuador, superpáramo; (f) *Senecio nivalis*, Ecuador, superpáramo, (g) *S. pindilicensis*, Ecuador, montane forest; (h) *S. patens,* Ecuador, montane forest. Symbols are colored according to species assignment to the main structure clusters (see Figure 2); symbol shape indicates the growth form, that is, square–basal rosette herb, circle–narrow‐leaved subshrub, triangle–broad‐leaved liana

Species of the genus *Senecio* L. (Asteraceae), which were traditionally placed in the genus *Lasiocephalus* Willd. ex Schltdl. (Cuatrecasas, 1978), comprise a morphologically and ecologically diverse plant group in the northern and central Andes. About 25 species are distributed from Venezuela to Bolivia, with the highest richness in Ecuador (Calvo & Freire, 2016; Cuatrecasas, 1978). Two main growth forms are recognized. Broad‐leaved lianas (Figure 1 g,h) inhabit montane forests and secondary thickets usually between 2,800 and 3,800 m, although some species also occur in the forest–páramo shrubby ecotone called subpáramo (usually at 3,800 m). The other growth form is ascending or erect, narrow‐leaved subshrub (Figure 1a–c,e) that occur in the páramo dominated by tussock grasses (3,800–4,300 m) and in the uppermost belt of patchy vegetation called superpáramo (up to 4,800–5,000 m). One species, *Senecio mojandensis* Hieron. (Figure 1d), a basal rosette herb of wet páramo habitats cannot be satisfactorily placed in either of these categories. Most species are morphologically distinct and readily identifiable, although some are variable in leaf size and shape, such as *S. otophorus* Wedd.

Phylogenetic molecular studies of the tribe *Senecioneae* suggest that the traditionally recognized Andean genera *Lasiocephalus* and *Culcitium* Bonpl. (scapose herbs forming basal leaf rosettes) belong to *Senecio* (Pelser, Nordenstam, Kadereit, & Watson, 2007; Pelser et al., 2010). Our previous study of 13 *Senecio* species from the former *Lasiocephalus*, which all were diploid, based on nuclear DNA sequences (ITS region) and nuclear genome size data (Dušková et al., 2010), identified two major clades that largely correspond to the two habitat types, that is, montane forest and páramo. The results also suggested that *Senecio* (*Culcitium*) *nivalis* (Kunth) Cuatrec. (Figure 1f) was closer to species of former *Lasiocephalus* than to other taxa of former *Culcitium*.

Given its likely origin within ca. the last 2 Myr (Pelser et al., 2010) and occurrence in the montane‐alpine habitats, the former *Lasiocephalus* exemplifies recent plant radiation in the (sub)tropical Andes. Based on extensive population sampling throughout the northern Andes and using an extended sample of ITS sequences complemented with highly variable AFLP (amplified fragment length polymorphism) markers, here we present deeper insights into the relationships among the Andean species of *Senecio* formerly classified in *Lasiocephalus*. Specifically, we examine a hypothesis put forward by Dušková et al. (2010) that independent transitions between the montane forest and páramo habitats occurred that were accompanied by growth form changes. We further examine patterns of genetic diversity within the group, and particularly their correlation with environmental factors and Andean geography.

## 2 MATERIALS AND METHODS

### 2.1 Plant material

**Figure 2:**
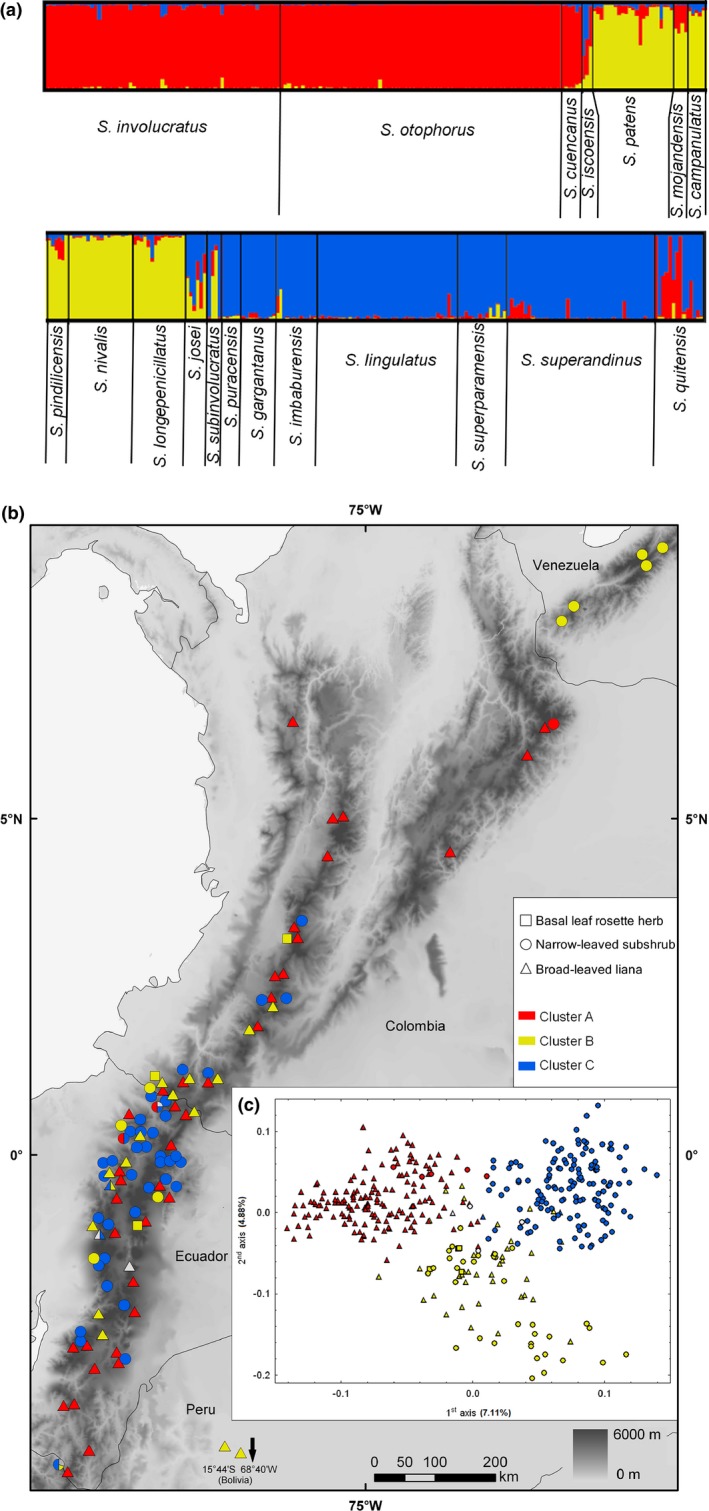
(a) Assignment of 374 individuals (entire dataset) of high‐elevation Andean *Senecio* into three main AFLP clusters inferred in structure; (b) Geographical locations of populations with growth form and structure cluster assignment indicated; (c) Ordination of AFLP phenotypes by use of principal coordinate analysis (PCoA) based on Jaccard distances. The symbol coloration reflects the assignment of the individuals to the main structure clusters (white–admixed individuals with assignment probability below 0.5); symbol shape indicates the growth form, that is, square–basal rosette herb, circle–narrow‐leaved subshrub, triangle–broad‐leaved liana

Samples of species from the former *Lasiocephalus* and former *Culcitium*, along with co‐occurring species of *Senecio*, were collected during 2006–2010 in Bolivia, Ecuador, Venezuela, and Colombia (Appendix S1). Due to the sampling gap in the central Andes, we lacked the single Peruvian species of former *Lasiocephalus*, a broad‐leaved liana *S. loeseneri* Hieron. This species is, nevertheless, sometimes considered conspecific with *S. campanulatus* Sch. Bip. ex Klatt from Bolivia (Calvo & Freire, 2016), which was included in our study. Multiple populations were sampled for most of the species throughout their distribution ranges (Figure 2b). At each locality, geographical coordinates and elevation were recorded. Young, intact leaves were collected and desiccated in silica gel; vouchers were deposited in COL, PRC, QCA, QCNE, and VEN.

### 2.2 AFLP fingerprinting and DNA sequencing

In total, 356 accessions of 18 *Senecio* species formerly classified as *Lasiocephalus* and 18 accessions of *Senecio nivalis* were genotyped using AFLP fingerprinting (Vos et al., 1995) (see Appendix S2 for details on the protocol). Fragments were manually scored with genemarker version 1.80 (SoftGenetics). Only unambiguous fragments in the range of 60‐500 bp. were scored, regardless of their intensity (Tribsch, Schönswetter, & Stuessy, 2002). For 5% of the samples, the whole AFLP protocol was repeated from the isolated DNA onwards to test the reproducibility of the method (Bonin et al., 2004). Internal transcribed spacer (ITS) regions were directly sequenced using the primers ITS4 and ITS5 (White, Bruns, Lee, & Taylor, 1990) for 50 individuals of Andean *Senecio* (i.e., 44 of former *Lasiocephalus*, two of former *Culcitium*, four other members of *Senecio*). We selected the individuals in order to representatively cover all species of the former *Lasiocephalus* as well as all clusters and subgroups identified by AFLPs.

### 2.3 Clustering of AFLP data

Genetic structure was inferred using a Bayesian clustering method implemented in structure 2.2.3 (Falush, Stephens, & Pritchard, 2007) employing a recessive allele model with admixture, assuming independent allele frequencies with 1,100,000 MCMC (Markov chain Monte Carlo) generations, and discarding the first 100,000 generations as burn‐in. We limited the number of clusters (*K*) to 1–10, each *K* was replicated with ten runs, and we further assessed stability of the results by calculating similarity coefficients between the replicate runs (Nordborg et al., 2005) and delta *K* (Evanno, Regnaut, & Goudet, 2005), both calculated using the R‐script Structure‐sum‐2009 (Ehrich, 2006). The Ks with consistent results over ten repeats were considered to be plausible and further examined. As the analysis of the entire dataset showed that only runs with *K* = 3 converged to a consistent solution in ten repeats, subsequent, separate structure analyzes of each of these three partitions (hereafter named clusters A, B, and C) were conducted using the same parameters. Only individuals assigned to a particular cluster with posterior probability >0.9 in the initial analysis were included in these subsequent analyzes. Major trends in the AFLP variation were visualized using principal coordinate analyzes (PCoA) based on Jaccard interindividual distances computed using famd 1.31 (Schlüter & Harris, 2006).

We further investigated the relationships among the major clusters based on a reduced subset of 266 individuals that were identified as nonadmixed (i.e., with posterior probabilities of membership to both major clusters and subgroups >0.9) in the structure analyzes. We reconstructed phylogenetic relationships using a likelihood model for binary restriction site data implemented in mrbayes v3.2.5 (Ronquist & Huelsenbeck, 2003). This model approximates the gain and loss of fragments by setting a condition that the characters that are absent (i.e., 0) in all individuals cannot be observed. We performed two independent runs of 5,000,000 generations each using the default prior settings, setting the restriction site model (lset nst = 1 coding = noabsencesites) and discarding the first 25% generations as burn‐in.

### 2.4 DNA sequence analyzes

Sequences of the ITS region were aligned by mafft 7 (Katoh & Standley, 2013) and edited using aliview (Larsson, 2014). In addition, we included in the final matrix previously published ITS sequences of: (1) 11 directly sequenced accessions of other Andean *Senecio* (Pelser et al., 2007) and (2) 26 cloned individuals from the former *Lasiocephalus* (10–12 colonies per accessions were cloned; putative PCR errors and potential chimaeric sequences were removed previously by Dušková et al., 2010; no excessively long branches indicating nonfunctional copies were found). To reduce the number of phylogenetically uninformative tip branches (and number of pseudoreplicates for trait mapping analyzes), we collapsed the cloned sequences from each individual to a consensus and intra‐individual polymorphisms were coded using IUPAC (International Union of Pure and Applied Chemistry) ambiguity codes in cases where clones from single species formed a monophyletic cluster or fell within an unresolved polytomy. The single exception with highly divergent haplotypes was 88_Pi, in which the two divergent haplotypes were retained as separate accessions when constructing the tree. We performed phylogenetic analysis on the resulting matrix of 630 characters and 87 individuals using both maximum parsimony (in paup v4.0b10; Swofford, 2002; treating gaps as characters) and Bayesian analyzes (in mrbayes v3.2.2; Ronquist & Huelsenbeck, 2003). The most parsimonious trees were searched heuristically with 1,000 replicates of random sequence addition, tree bisection reconnection swapping and MulTrees on and the data set were bootstrapped using 1,000 replicates. In Bayesian analyzes, we applied the generalized time reversible (GTR) substitution model (as selected by the Bayesian Information Criterion in JModeltest 2; Darriba, Taboada, Doallo, & Posada, 2012) with gamma distribution of rate heterogeneity and simultaneously ran two MCMCMC runs with four chains each for 2,000,000 generations, sampling every 1000th generation using the default priors. The posterior probability of the phylogeny and its branches were determined from the combined set of trees, discarding the first 25% of trees as burn‐in.

### 2.5 Growth form evolution

Character state reconstructions of the growth forms were performed employing a maximum likelihood approach implemented in the function rayDISC, part of the package corHMM (Beaulieu, O'Meara, & Donoghue, 2013) in R (Ihaka & Gentleman, 1996). This method allows for reconstructions of multistate characters, unresolved nodes, and ambiguities (polymorphic taxa or missing data). Three models of character evolution were evaluated as follows: equal rates (ER), symmetrical (SYM), and all rates different (ARD); an Akaike information criterion corrected for sample size (AICc) was used to select the best fitting model. Association of growth forms and phylogeny was tested by computing Pagel's lambda (Freckleton, Harvey, & Pagel, 2002) using the function fitDiscrete in the package geiger (Pennell et al., 2014) in R (Ihaka & Gentleman, 1996). Statistical significance of estimated lambda was tested by computing likelihood ratio test (LRT) against lambda = 0 model.

### 2.6 Geographical analyzes of AFLP data

Geographical correlates of the genetic (AFLP) variation were examined after the admixed (i.e., posterior probability <.9), and Bolivian samples were excluded to avoid bias due to unclear cluster assignment and sampling gap, respectively. We tested for a significant correlation between matrices of genetic and geographical distances among populations (isolation by distance) using a Mantel test in *adegenet*. Among‐population genetic chord distances derived from AFLP fragment frequencies were inferred using a Bayesian method with nonuniform priors (Zhivotovsky, 1999) as implemented in famd 1.31 (Schlüter & Harris, 2006).

Climatic data describing mean annual temperature, daily and annual temperature ranges, annual rainfall, and its inter‐annual variation expressed as coefficient of variation for each collection site were extracted from the WorldClim database (Hijmans, Cameron, Parra, Jones, & Jarvis, 2005). Those data together with elevation formed a group of environmental variables, while site latitude and longitude represented geographical variables. Variance of the AFLP data matrix was partitioned into environmental and geographical components (and their interaction) by a series of redundancy analyzes (RDA) and partial RDA ordinations (Borcard, Legendre, & Drapeau, 1992) employing canoco for windows 4.5 (ter Braak & Šmilauer, 1998). As the RDA employs Euclidean distance to measure dissimilarity between pairs of samples (Šmilauer & Lepš, 2014), this makes it analogous to analysis of molecular variance (AMOVA; Excoffier, Smouse, & Quattro, 1992) but provides an opportunity to make partial tests to discriminate between pure effects of explanatory variables and their interaction.

## 3 RESULTS

### 3.1 AFLP fingerprinting

AFLP analysis of 374 accessions resulted in 269 reliable fragments, of which 264 (98%) were polymorphic. The overall reproducibility of the dataset was 95%.

#### 3.1.1 Main grouping within the entire dataset

Bayesian clustering of the entire dataset yielded the highest values of Δ*K* and among‐replicate similarity (1.0) for partition into three clusters A, B, and C (Figure 2a, Appendix S3A). Cluster A contained mostly lianas of montane forest and forest–páramo ecotone, one of which, however, also formed a morphologically distinct subshrub‐like high‐elevation population (pop. 51). Cluster B was morphologically variable, encompassing montane forest lianas*,* narrow‐leaved páramo subshrubs*,* and a basal rosette herb. Cluster C contained exclusively narrow‐leaved (super)páramo subshrubs (Figure 2b, Table 1). Four species comprised mostly individuals that were admixed either between all three clusters (*S. josei* Sklenář, *S. iscoensis* Hieron.), between clusters A and C (*S. *aff. *quitensis*), or between clusters B and C (*S. subinvolucratus* Cuatrec.), although certain admixture was also found in some individuals of *S. patens* (Kunth) DC., *S. mojandensis*,* S. pindilicensis* Hieron., *S. longepenicillatus* Sch. Bip. ex Sandwith, *S. imbaburensis* Sklenář & Marhold, *S. lingulatus* (Schltdl.) Cuatrec., and *S. superandinus* Cuatrec.. The Bayesian clustering was also reflected in PCoA ordination, separating the three clusters along the first (cluster A vs. C) and second (cluster B vs. A + C) axes (Figure 2c).

**Table 1:** Summary of the associations between growth forms (uppercase letters) and genetic relationships reconstructed by the two sets of molecular markers (AFLPs, ITS sequences) in high‐elevation north Andean *Senecio* (see Figures 2, 3, 4, 5 for AFLP (sub)groups and Figure 6a for ITS (sub)clades delimitation). B–basal rosette herb, mostly from lower páramo; L–broad‐leaved liana, from montane forest and forest‐páramo ecotone; N–narrow‐leaved subshrub, from páramo to superpáramo. Others refer to admixed samples with equivocal assignment to AFLP (sub)groups and accessions not genotyped by AFLPs (see Appendix S1)

	Páramo ITS clade		Forest ITS clade					
	p1	p2	f1	f2	f3	f4	f5	f6
AFLP cluster A								
A1								L
A2							L	
A3							L	N, L
AFLP cluster B								
B1	N							
B2					N			
B3						L		
B4						L		
B5			L					
AFLP cluster C								
C1		N		N				
C2		N						
C3	N	N						
C4	N	N						
C5		N						
Others	B	N, L		B		L	N, L	N

#### 3.1.2 Finer structure within the main clusters

**Figure 3:**
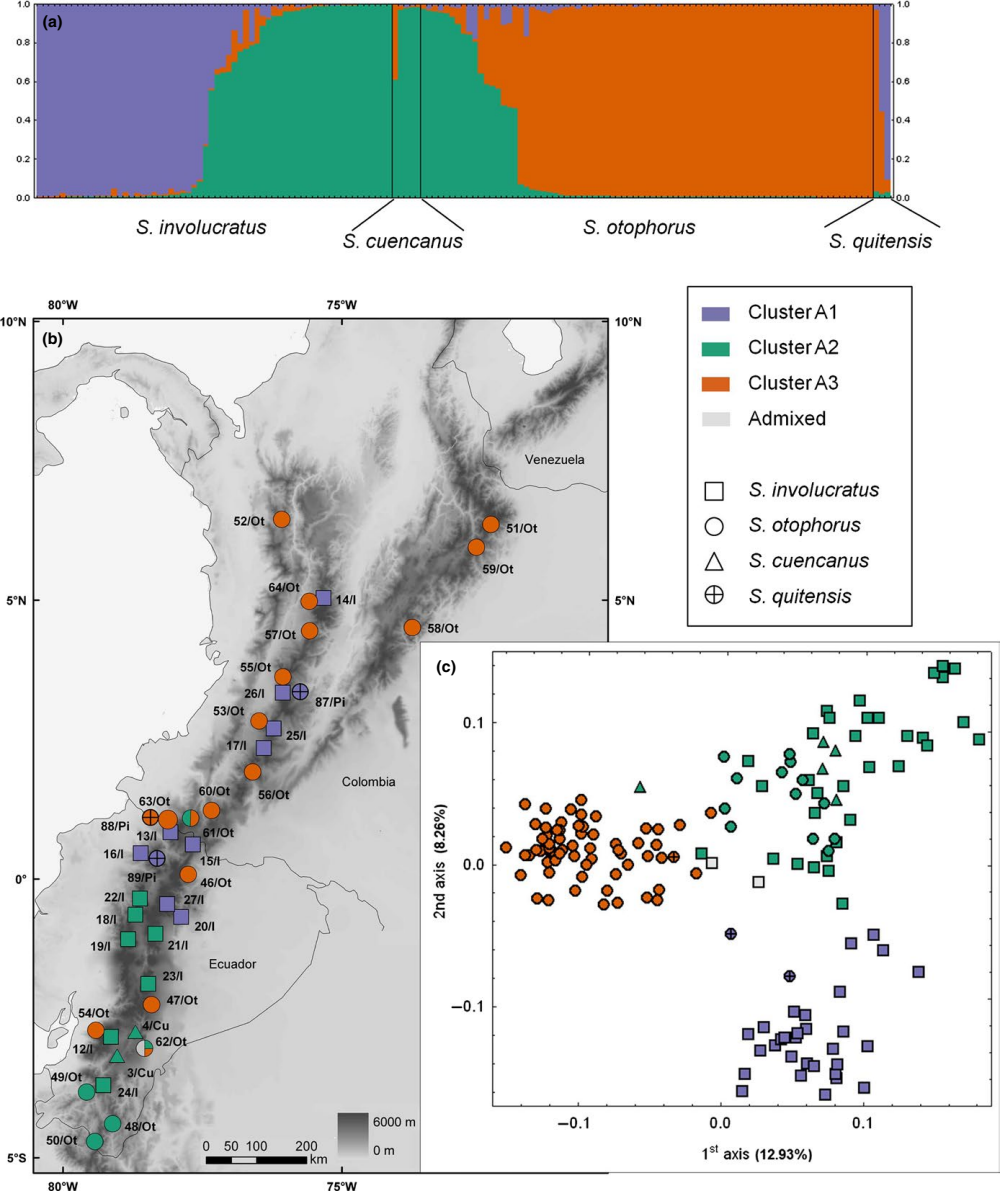
Genetic structure and geographical distribution of 149 individuals of high‐elevation Andean *Senecio* from cluster A. (a) Posterior probabilities for membership of each individual in the three resulting subgroups (designated by different colors) as identified in a separate structure analysis of cluster A members. (b) Geographical distribution of the analyzed populations. (c) Ordination of AFLP phenotypes (PCoA); symbol color refers to the structure subgroups (>0.5 posterior probability), symbol shape indicates species

Separate Bayesian clustering of the accessions assigned to cluster A revealed that *K* = 2 and *K* = 3 exhibited high similarity among independent runs (>0.998 in both partitions, although the former had higher Δ*K*; Appendix S3A), and the finer structuring was plotted onto the map (Figure 3b). With *K* = 2, *S. involucratus* and *S. cuencanus* Hieron. were classified in the first subgroup, although most of their accessions were admixed, while most accessions of *S. otophorus* (excluding those from southern Ecuador) fell into the second subgroup (Appendix S3B). With *K* = 3, two subgroups partly corresponded to species limits, namely (1) a subgroup of Colombian and north Ecuadorian populations of *S. involucratus* (Kunth) DC. (along with two *S. *aff. *quitensis* Cuatrec. accessions) and (2) a subgroup of Colombian to central Ecuadorian accessions of *S. otophorus*. The third subgroup comprised all populations from southern Ecuador disregarding species identity (*S. involucratus, S. cuencanus*, and *S. otophorus*) along with populations of *S. involucratus* from northern and central Ecuador (Figure 3a,b). The PCoA of cluster A confirmed the Bayesian grouping, separating Colombian to central Ecuadorian populations of *S. otophorus* along the first axis and Colombian to north Ecuadorian populations of *S. involucratus* along the second axis (Figure 3c).

**Figure 4:**
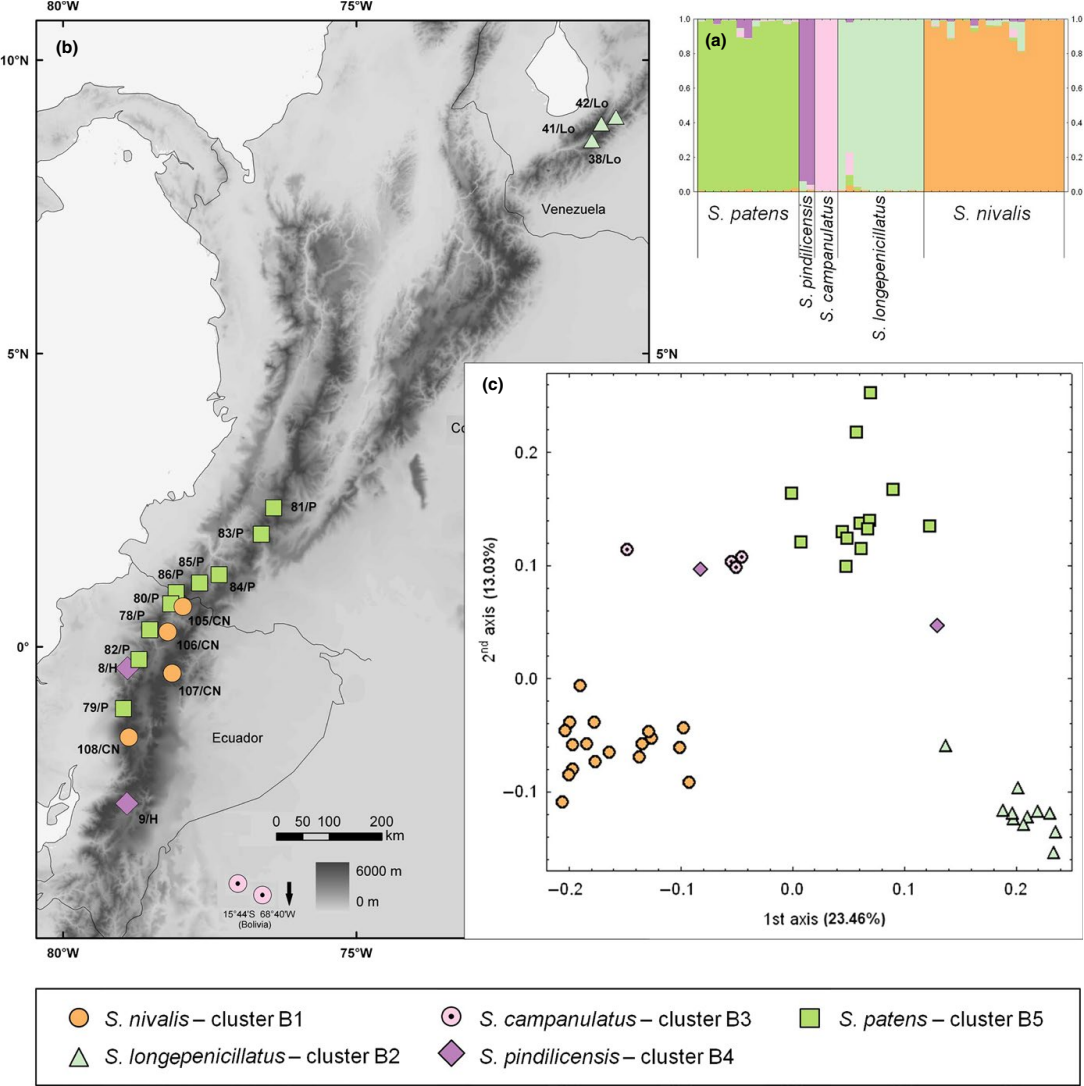
Genetic structure and geographical distribution of 47 individuals of high‐elevation Andean *Senecio* from cluster B. (a) Posterior probabilities for membership of each individual in the five resulting subgroups (designated by different colors) as identified in a separate structure analysis of cluster B members. (b) Geographical distribution of the analyzed populations. (c) Ordination of AFLP phenotypes (PCoA); symbol color refers to the structure subgroups (>0.5 posterior probability), symbol shape indicates species

Separate analysis of cluster B yielded the same similarity coefficient, 1.0, for *K* = 2, 3, 4, and 5, although *K* = 3 had the highest Δ*K* (Appendix S3A). The finest partitioning (*K* = 5) separated all five species with almost no admixture (Figure 4a), whereas *S. patens* and *S. pindilicensis* merged at *K* = 4, and *S. campanulatus* and *S. longepenicillatus* joined this subgroup at *K* = 3 and *K* = 2, respectively, leaving *Senecio nivalis* apart from all other species at *K* = 2 (Appendix S3C). The PCoA of cluster B (Figure 4c) separated *S. nivalis* and *S. patens* along the first two ordination axes, whereas the third and fourth axes separated *S. campanulatus* and *S. pindilicensis*, respectively.

**Figure 5:**
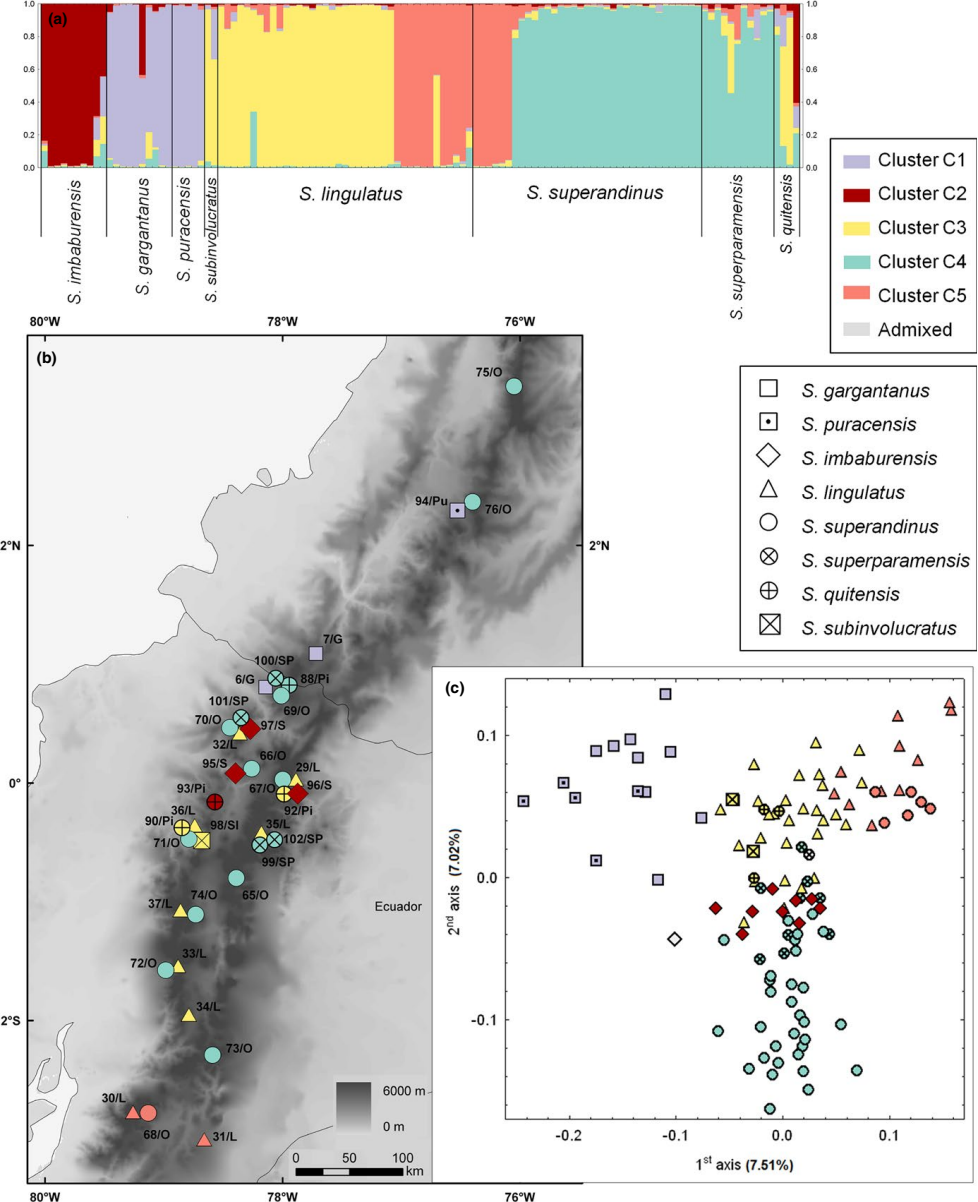
Genetic structure and geographical distribution of 116 individuals of high‐elevation Andean *Senecio* from cluster C. (a) Posterior probabilities for membership of each individual in the five resulting subgroups (designated by different colors) as identified in a separate structure analysis of cluster C members. (b) Geographical distribution of the analyzed populations. (c) Ordination of AFLP phenotypes (PCoA); symbol color refers to the structure subgroups (>0.5 posterior probability), symbol shape indicates species

Analysis of cluster C showed the highest Δ*K* and similarity coefficient (0.995) to be yielded by *K* = 5 (Appendix S3A). The five subgroups comprised as follows: (1) *S. puracensis* (Cuatrec.) Cuatrec. + *S. gargantanus* (Cuatrec.) Cuatrec.; (2) *S. imbaburensis*; (3) *S. lingulatus* (except for southern Ecuador) + *S. subinvolucratus*; (4) *S. superandinus* (except for southern Ecuador) + *S. superparamensis* Sklenář, (5) southern Ecuadorian populations of *S. superandinus* and *S. lingulatus* (Figure 5a,b). *Senecio* aff. *quitensis* was highly admixed and was scattered among three subgroups. The PCoA ordination diagram showed incomplete discrimination of the species (Figure 5c), but its first and second axes, respectively, suggested separation of the *S. gargantanus* + *S. puracensis* group and supported the distinct position of the southern Ecuadorian populations of *S. superandinus* and *S. lingulatus*.

### 3.2 ITS and AFLP phylogeny

**Figure 6:**
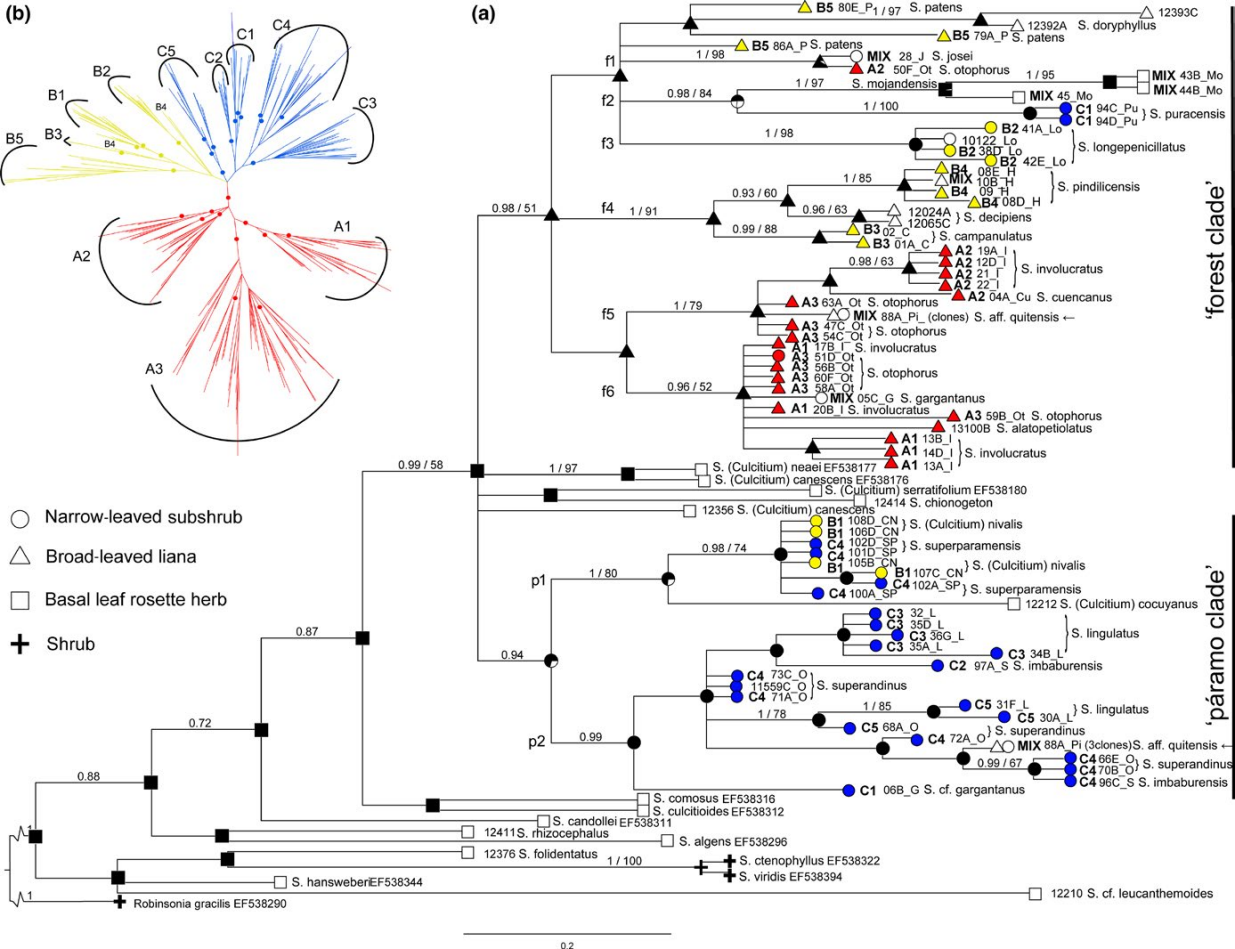
(a) Phylogenetic reconstruction of 87 accessions of northern Andean *Senecio* based on sequences of ITS region of ribosomal DNA. Bayesian 50% majority rule consensus tree with posterior probabilities >0.90 and bootstrap values >50% inferred with maximum parsimony are indicated, respectively, before and after the slash above each supported branch. Supported subclades of the “forest” and “páramo” clades are marked as f1–f6 and p1–p2, respectively. Growth form of each accession is marked by a symbol, membership in the AFLP subgroups (if applicable) is denoted by corresponding letters (A1–C5), accessions with ambiguous structure assignment are marked “MIX.” The presence of highly divergent ITS sequences in the same individual of *S. *aff. *quitensis* is marked by an arrow. *Senecio doryphyllus*,* S. decipiens,* and *S. alatopetiolatus*, although belonging to the former *Lasiocephalus*, were not analyzed using the AFLPs. Reconstruction of the growth form evolution according to the equal rates (ER) model has been superimposed onto the ITS tree (see Appendix S3E for original). (b) Relationships among AFLP phenotypes of 266 nonadmixed (see Section 2) individuals of former *Lasiocephalus* and *Senecio nivalis* reconstructed in Bayesian framework. Cluster codes correspond with Figures 3, 4, 5; branches with posterior probabilities >0.95 are marked with dots

Bayesian analysis of ITS sequences showed monophyly of a clade comprising all accessions of the former *Lasiocephalus* and former *Culcitium* (together with *Senecio chionogeton*), although it did not support separation of the two former genera (Figure 6a). Instead, along with several unresolved former *Culcitium* accessions, we identified two clades, corresponding to “páramo” and “forest” clades of Dušková et al. (2010), which with a few exceptions corresponded to major AFLP clusters C and A + B, respectively (Figure 6a,b, Table 1). The “forest clade” further split into several well supported subclades (with uncertain relationships among them) which mostly corresponded to AFLP subgroups (namely B2, B3 + B4, B5, A1 + A3, A2 + A3 subgroups). While the “páramo clade” comprised narrow‐leaved subshrubs and one basal rosette herb, the “forest clade” contained representatives of all three growth forms. Broad‐leaved liana is reconstructed as the ancestral state within the “forest clade,” which contains one subclade (f4) formed by lianas only, three subclades (f1, f5, f6) comprising a liana and a narrow‐leaved subshrub, one subclade (f3) with a narrow‐leaved subshrub (*S. longepenicillatus*), and one subclade (f2) comprising a narrow‐leaved subshrub and a basal rosette herb (Table 1).

There were several remarkable incongruences among ITS and AFLP data. In particular, *Senecio nivalis* (cluster B1) shared the same ITS haplotypes with *S. superparamensis* (cluster C4) and both species formed a supported lineage (p1) within the “páramo” clade (Figure 6a). AFLP cluster C1 was split into both major ITS clades, with the Colombian and Ecuadorian accessions being parts of the “forest” and “páramo” clades, respectively. ITS clones isolated from a single accession of *S. *aff. *quitensis* (pop. 88_Pi) fell into both major ITS clades. Finally, *S. puracensis* from “páramo” cluster C appears nested within the ITS “forest clade.”

Bayesian phylogenetic analysis of AFLP phenotypes of nonadmixed individuals confirmed monophyly (>90% posterior probability) of most of the AFLP subgroups. However, it failed to provide support for relationships among the AFLP subgroups, except for supported monophyly of cluster A (Figure 6b, Appendix S3D).

### 3.3 Growth form evolution

Equal rates (ER) model had the lowest AICc (94.24), compared to SYM (97.77) and ARD (107.13) models. The ER model reconstructed the basal rosette herb as the ancestral growth form for the *Lasiocephalus*‐*Culcitium* species group (Figure 6a, Appendix S3E). The herbs switched to broad‐leaved lianas in the “forest clade,” with further changes to narrow‐leaved subshrub (*S. longepenicillatus*,* S. puracensis*) and a reversal to a basal rosette herb (*S. mojandensis*). The growth form of narrow‐leaved subshrub is present in all species of the “páramo clade” except for *S. cocuyanus* (basal rosette herb) and *S. *aff. *quitensis* (varying between broad‐leaved liana and narrow‐leaved subshrub). The growth form was strongly associated with phylogeny (Pagel's lambda = 0.89, ln(lambda) = −75.63, ln(lambda = 0) = −150.38, LRT *p*‐value < .001).

### 3.4 Geographical and environmental analyzes of AFLP data

*Senecio* populations as a whole and members of cluster A showed a very weak correlation between genetic and geographical distances (isolation by distance, IBD), whereas this relationship was nonsignificant in the other two clusters (Table 2). Subgroups with sufficient numbers of populations were available only in cluster A; here we observed significant correlations in both Ecuadorian‐Colombian subgroups A1 and A2 but a lack of correlation in the southern Ecuadorian subgroup A2.

**Table 2:** Eco‐geographical covariates of genetic variation of north‐Andean *Senecio*. Variance partitioning (by means of RDA) of AFLP genetic variation into environmental (rainfall, temperature, elevation) and geographical (latitude, longitude) components and correlation between genetic and geographical distances (by means of Mantel test and quantified by correlation coefficient *r*_M_). The partitioning was based on characteristics of the original collection sites after genetically admixed individuals, and spatially remote Bolivian samples were excluded; all RDA ordinations were significant at *p* = .002 under 499 Monte Carlo permutations

	Entire dataset	Cluster A	Cluster B	Cluster C
Variance partitioning (RDA)				
Environment	5.8%	7.8%	31.2%	8.8%
Environment^a^ geography	1.5%	5.2%	0%	1.1%
Geography	2.2%	6%	7.7%	5.2%
Residual	90.5%	81%	61.1%	84.9%
Isolation by distance (Mantel test)	*r*_M_ = .12, *p* = .04	*r*_M_ = .12, *p* = .04^a^	n.s.	n.s.

a Subgroups, A1: *r*_M_ = .44, *p* = .02; A2: n.s.; A3: *r*_M_ = .31, *p* = .03.

Less than 10% of total variance in the entire AFLP dataset was accounted for by the effects of either environmental (rainfall, temperature, elevation; altogether 6% of variability) geographical (latitude, longitude; 2% of variability) components or their interaction (1.5% of variability) (Table 2). When the three AFLP clusters were analyzed separately, the geographical component accounted for a similar (5%–8%) proportion of the total variation, whereas the environmental component accounted for almost a third of variation in cluster B but only 8%–9% in clusters A and C. Moreover, there was a distinct interaction (5%) between the two sets of variables in cluster A, whereas the interaction was very low or lacking in the two other clusters.

## 4 DISCUSSION

### 4.1 *Lasiocephalus*‐*Culcitium* species group

Pelser et al. (2007, 2010) and Dušková et al. (2010) pointed to close relationships between the former genera of *Lasiocephalus* and *Culcitium*. The present study, using ITS sequences and an extended list of species, suggests monophyly of the *Lasiocephalus*‐*Culcitium* species group but with neither of the two former genera monophyletic. Although relationships within the group are only partly resolved in both the ITS and AFLP datasets, suggesting a recent diversification (Turner et al., 2013), there is a partial congruence between the two markers, as AFLP cluster C corresponds to the ITS “páramo clade” (except for *Senecio nivalis*) and AFLP clusters A and B correspond to the ITS “forest clade” (except for *S. puracensis*) (Figure 6, Table 1); incongruences will be discussed below.

### 4.2 Growth form changes and habitat shifts

Species of different growth forms and preferences for páramo or montane forest fell within several different AFLP (sub)groups and ITS (sub)clades, suggesting that independent shifts in ecology were accompanied by changes in morphology. Both AFLP and ITS data indicate that at least two distinct genetic entities occur in the páramo, representatives of which demonstrate convergence in such traits as growth form, size and number of capitula, and leaf morphology (Figure 1). The first entity is the páramo‐dwelling AFLP cluster C (largely corresponding to the ITS “páramo clade”), species of which occur throughout most of Ecuador and southern Colombia. The second entity is represented by Venezuelan *S. longepenicillatus* (AFLP cluster B, f3 subclade within the ITS “forest clade”), which is a narrow‐leaved páramo subshrub but sporadically also appears in a broad‐leaved form at the tree‐line ecotone. In addition, *S. otophorus* (AFLP cluster A, f6 subclade within the ITS “forest clade”), which grows as a slender liana twining in subpáramo thickets, also occurs as a narrow‐leaved subshrub in the superpáramo of Colombian Cordillera Oriental (Figure 1c), and similar habit variation is demonstrated by montane forest and superpáramo plants of *S. doryphyllus* Cuatrec. from Sierra Nevada de Santa Marta (northern Colombia). These findings suggest that convergent growth form evolved independently in various parts of the northern Andes, although the variation in *S. otophorus* and *S. doryphyllus* may only represent phenotypic plasticity.

Transitions in growth form associated with habitats at different elevations have been presented for various Andean plant groups. Whereas Lobeliaceae (Knox, Muasya, & Nuchhaala, 2008), *Huperzia* Bernh. (Wilkström, Kenrick, & Chase, 1999), *Chusquea* Kunth (Fisher et al., 2009), and *Disterigma* (Klotzsch) Nied. (Pedraza‐Peñalosa, 2009) apparently colonized alpine habitats from the montane forest, for *Chaetanthera* Ruiz & Pav. and *Puya* Molina migration was suggested in the opposite or both directions, respectively (Hershkovitz, Arroyo, Bell, & Hinojosa, 2006; Jabaily & Sytsma, 2012). As a grade of herbaceous *Senecio* species from alpine habitats subtends (although the support is weak) the *Lasiocephalus*‐*Culcitium* clade (Figure 6a), the ITS phylogeny is consistent with an alpine‐to‐forest transition for the evolution of the *Lasiocephalus*‐*Culcitium* group as a whole. If such a relationship is confirmed, a change from the herbaceous (basal leaf rosette) to the woody (liana, ascending subshrub, shrub) state is implied, similar to, for example, Andean *Valeriana* L., *Gentianella* Moench, and *Loricaria* Wedd. (Kolář, Dušková, & Sklenář, 2016; Sklenář et al., 2011). The polytomy consisting of the “páramo clade,” “forest clade,” and several basal rosette herbs does not permit interpretation of the growth form transitions within the *Lasiocephalus*‐*Culcitium* clade to evaluate Cuatrecasas' (1978) idea that the páramo growth form of former *Lasiocephalus* species evolved from the growth form of their montane forest ancestor(s). However, Cuatrecasas' view could be valid for some páramo subshrubs found within the “forest clade” (e.g., *S. longepenicillatus*). Moreover, the ITS phylogeny suggests another transition in this clade, that is, to a basal rosette herb in *S. mojandensis*.

### 4.3 Role of hybridization

Gene flow is known to occur among closely related alpine species (Vargas, 2003; Wagstaff & Garnock‐Jones, 2000; Winkworth, Wagstaff, Glenny, & Lockhart, 2005), yet the role of hybridization in the evolution of the Andean flora has not been documented, except in the cases of *Polylepis* Rioz & Pav. (Schmidt‐Lebuhn, Kessler, & Kumar, 2006), *Hypochaeris* (Tremetsberger et al., 2006), and *Puya* (Jabaily & Sytsma, 2012). In agreement with frequent hybridization in the *Senecioneae* (Hodálová & Marhold, 1996; Kirk, Máčel, Klinkhamer, & Vrieling, 2004; Lowe & Abbott, 2000; Osborne, Chapman, Nevado, & Filatov, 2016), our molecular data, morphological observations, and previously published genome size values (Dušková et al., 2010) indicate that homoploid hybridization likely occurred among multiple species of former *Lasiocephalus*.

Traces of hybridization are indicated by consistently admixed AFLP profiles across multiple populations of several species (Figure 2a). This is especially apparent for *Senecio* aff. *quitensis*, a taxon with morphology varying between subshrubs and lianas and whose accessions variously combine AFLP profiles of clusters A (forest lianas) and C (páramo subshrubs). Furthermore, ITS sequences of this species (including divergent ITS copy types from a single individual) are placed in the divergent “páramo” and “forest” clades, and its genome size is intermediate between them (Dušková et al., 2010).

Incongruence between the AFLP and ITS datasets suggests that hybridization might also have been involved in the origin of other Andean *Senecio* species. This was particularly documented for *Senecio superparamensis*, a species of intermediate morphology and genome size (Dušková et al., 2010; as “*L*. sp. 4” there) between *S. superandinus*, with which it was assigned to AFLP subgroup C4, and *S. nivalis*, with which it shares ITS sequences (Figure 6a). Such conflict may be explained by (past) gene flow of *S. nivalis* ITS haplotypes that was followed by rapid homogenization of the ITS sequences (Alvarez & Wendel, 2003) toward *S. nivalis*‐like paralogues. A similar process might have led to “deeper” incongruences in other species (*S. nivalis* and *S. puracensis*; Figure 6a) that also exhibit conflicts among the three major AFLP clusters versus the two main ITS clades. Such indications of past hybridization events, however, should be interpreted with caution as AFLP data do not allow distinguishing admixture from incomplete lineage sorting.

### 4.4 Geographical and ecological correlates of genetic variation

Geographical barriers along with ecological differentiation promote species diversification in mountains (Kolář et al., 2016; Luo et al., 2016). As a geographical signal was comparably strong in the three main AFLP clusters, geography may structure genetic variation in a similar way in both the Andean montane forest and the páramo. In support of this, the AFLP data reveal two strikingly similar cases of genetic separation corresponding to geography which are incongruent with morphology‐based species limits. Two páramo species, *Senecio lingulatus* and *S. superandinus* (cluster C), are readily distinguished morphologically (Figure 1a,e) and are genetically distinct throughout most of Ecuador. Their populations in southern Ecuador, however, merge and form another distinct genetic subgroup (Figure 5a,b). Similarly, two morphologically distinct species from the montane forest‐páramo ecotone, *S. involucratus* and *S. otophorus* (cluster A), appear as distinct AFLP entities in northern‐central Ecuador and Colombia, but the markers fail to discriminate between them in southern Ecuador (Figure 3a,b). There, the species form a separate subgroup together with another morphologically distinct montane forest liana, *S. cuencanus*.

As morphologically intermediate plants between *S. superandinus* and *S. lingulatus* occur in southern Ecuador, gene flow due to hybridization might have generated the observed pattern. In contrast, we did not observe any putative hybrids between *S. involucratus* and *S. otophorus*, nor did we find any consistent morphological distinction in plants of either species from southern Ecuador. Therefore, we hypothesize that southern Ecuador represents an ancestral area of cluster A where high levels of ancestral polymorphisms have been retained, preventing the genetic discrimination of species by means of the AFLPs. Both species might have independently migrated northwards, leaving a footprint of gradual genetic differentiation which is documented by a significant isolation by distance relationship observed in A1 and A3 subgroups. Such northward migration would be consistent with the biogeographical reconstructions of Andean plant groups such as *Azorella*,* Oreobolus* R. Br., and *Puya* (Andersson, Kocsis, & Eriksson, 2006; Chacón, Madriñán, & Bruhl, 2006; Jabaily & Sytsma, 2012). The northern and central Ecuadorian Andes experienced a different Quaternary history from the south of the country, namely in having volcanism and glaciation (Jørgensen & Ulloa, 1994). Glaciation events and volcanism may have structured the genetic patterns of the species through the effects of repeated bottlenecks and founder events (Luo et al., 2016; Vásquez, Balslev, Hansen, Sklenář, & Romoleroux, 2016).

The genetic structure of species from the montane forest‐páramo ecotone (cluster A) and páramo (cluster C) showed little association with environmental variables, which we acknowledge might be at least partly due to the lack of precision of extrapolated climatic variables for high mountains (Hijmans et al., 2005; Kirchheimer et al., 2016). However, in cluster B, the high proportion of genetic variation associated with environmental factors is consistent with the variety of habitats occupied by its species. The ecological differentiation coupled with high AFLP and morphological diversification may suggest a relatively long divergence time and/or efficient isolation (Kolář et al., 2016). The very small or entirely lacking association between environment and geography in clusters C and B, respectively, suggests that two distinct and (largely) independent signals are involved. However, the stronger association in cluster A suggests that migration along the cordilleras was coupled with a shift in species ecology, such as the entry of *S. otophorus* in the superpáramo in Colombia.

## CONFLICT OF INTEREST

None Declared.

## AUTHOR CONTRIBUTIONS

All co‐authors participated in the field work and writing. Specifically, E.D. did most of the molecular work. F.K. lead analyzes of molecular data. P.S. conceived the ideas. D.V. participated in the molecular work. T.F. did the character state reconstruction. K.R. arranged for research permits in Ecuador. K.M. supervised the research.

Editor: Şerban Procheş
