# Supplementary material for: Growth form evolution and hybridization in Senecio (Asteraceae) from the high equatorial Andes
Source: Ecol Evol. 2017 Jul 10;7(16):6455–68. doi: 10.1002/ece3.3206 (PMC5574811; doi:10.1002/ece3.3206)
Supplement: Supplementary file 1 [file ECE3-7-6455-s001.doc]

*Journal of Biogeography*

**SUPPORTING INFORMATION**

**Article title** : Growth-form evolution and hybridization in *Senecio* (Asteraceae) from the high equatorial Andes

Authors: Dušková et al.

Appendix S1. List of species with the most commonly used synonyms in the former genera *Lasiocephalus* and *Culcitium* and the localities with details and numbers of individuals analyzed using AFLPs and ITS; BL refers to broad-leaved, NL refers to narrow-leaved.

Senecio alatopetiolatus J. Calvo, E. Freire & Sklenář

Senecio campanulatus Sch. Bip. ex Klatt [*Lasiocephalus campanulatus (Sch. Bip. ex Klatt) Cuatrec.*]

Senecio canescens (Bonpl.) Cuatrec. [Culcitium canescens Bonpl.]

Senecio cocuyanus (Cuatrec.) Cuatrec [Culcitium cocuyanum Cuatrec.]

Senecio cuencanus Hieron. [*Lasiocephalus cuencanus (Hieron.) Cuatrec.*]

Senecio decipiens Benoist [Lasiocephalus decipiens (Benoist) Cuatrec.]

Senecio doryphyllus Cuatrec. [Lasiocephalus doryphyllus (Cuatrec.) Cuatrec.]

Senecio gargantanus (Cuatrec.) Cuatrec. [*Lasiocephalus gargantanus (Cuatrec.) Cuatrec.*]

Senecio imbaburensis Sklenář & Marhold [*Lasiocephalus sodiroi (Hieron.) Cuatrec.*]

Senecio involucratus (Kunth) DC. [*Lasiocephalus involucratus (Kunth) Cuatrec.*]

Senecio iscoensis Hieron.

Senecio josei Sklenář

Senecio lingulatus (Schltdl.) Cuatrec. [*Lasiocephalus lingulatus Schltdl.*]

Senecio longepenicillatus Schultz-Bip. ex Sandw. [*Lasiocephalus longipenicillatus (Schultz-Bip. ex Sandw.) Cuatrec.*]

Senecio mojandensis Hieron. [*Lasiocephalus mojandensis (Hieron.) Cuatrec.*]

Senecio nivalis Kunth [*Culcitium nivale* (Kunth) Cuatrec.]

Senecio otophorus Wedd. [*Lasiocephalus otophorus (Wedd.) Cuatrec.*]

Senecio patens (Kunth) DC. [*Lasiocephalus patens (Kunth) Cuatrec.*]

Senecio pindilicensis Hieron. [ *Lasiocephalus heterophyllus (Turcz.) Cuatrec.*]

Senecio puracensis (Cuatrec.) Cuatrec. [*Lasiocephalus puracensis (Cuatrec.) Cuatrec.*]

Senecio quitensis Cuatrec. [*Lasiocephalus pichinchensis (Cuatrec.) Cuatrec.*]

Senecio subinvolucratus Cuatrec. [*Lasiocephalus subinvolucratus (Cuatrec.) Cuatrec.*]

Senecio superandinus Cuatrec. [*Lasiocephalus ovatus Schltdl.*]

Senecio superparamensis Sklenář

| Species | Population code | Growth form | No. of AFLP samples | ITS sample | Locality | Latitude | Longitude | Collector(s) | Voucher number |
| --- | --- | --- | --- | --- | --- | --- | --- | --- | --- |
| *S. campanulatus* | 1_C | BL liana | 1 | 1 | Bolivia: La Paz, east slopes of Sorata (3 300 m a.s.l.) | S 15.7703 | W 68.6305 | J. Macek | LC 1 |
| *S. campanulatus* | 2_C | BL liana | 4 | 1 | Bolivia: La Paz, north slopes of Sorata (3 300 m a.s.l.) | S 15.745 | W 68.6703 | J. Macek | LC3 |
| *S. cuencanus* | 3_Cu | BL liana | 5 | –– | Ecuador: Azuay, along the road from Cuenca to the Tinajillas pass (3 400 m a.s.l.) | S 3.1711 | W 79.0341 | P. Sklenář & J. Karbulková | 11128 |
| *S. cuencanus* | 4_Cu | BL liana | 1 | 1 | Ecuador: Azuay, along the road Gualaceo-Limon, montane forest and shrubby thickets (3 000 m a.s.l.) | S 2.9333 | W 78.7001 | P. Sklenář & J. Karbulková | 11167 |
| *S. gargantanus* | 5_G | NL ascending subshrub | 2 | 1 | Colombia: Nariňo, Volcan Galeras, road from Pasto to the crater (3 860 m a.s.l.) | N 1.2214 | W 77.3447 | P. Sklenář & E. Dušková | 12391 |
| *S.* cf. *gargantanus* | 6_G | NL ascending subshrub | 5 | 1 | Ecuador: Carchi, Volcan Chiles, margin of the road Tufino - Maldonado, ca 100 m E of Laguna Verde (4 030 m a.s.l.) | N 0.8006 | W 77.9358 | P. Sklenář, E. Rejzková, F. Kolář | 11511 |
| *S. gargantanus* | 7_G | NL ascending subshrub | 5 | –– | Colombia: Nariňo, Volcan Azufral, along the path from Tuquerres to Laguna Verde, ca 300 m E of the laguna (3 970 m a.s.l.) | N 1.0913 | W 77.7154 | F. Kolář | 54 |
| *S. pindilicensis* | 8_H | BL liana | 2 | 2 | Ecuador: Pichincha, along the road Quito-Nono (3 250 m a.s.l.) | S 0.0963 | W 78.5562 | P. Sklenář & A. Kučerová | 11101 |
| *S. pindilicensis* | 9_H | BL liana | 1 | 1 | Ecuador: Azuay, thickets along the road between Canad and Azogues (2 890 m a.s.l.) | S 2.6708 | W 78.9061 | P. Sklenář & A. Kučerová | 11115 |
| *S. pindilicensis* | 10_H | BL liana | 3 | 1 | Ecuador: Chimborazo, along the road between Chunchi and Zhud, near Sta. Rosa (2 840 m a.s.l.) | S 2.3631 | W 78.97 | P. Sklenář & J. Karbulková | 12001 |
| *S. involucratus* | 11_I | BL liana | 2 | –– | Ecuador: Pichincha, along the road from San Juan towards the antennas de Atacazo (3 440 m a.s.l.) | S 0.2885 | W 78.6247 | P. Sklenář, A. Kučerová & P. Macek | 11065 |
| *S. involucratus* | 12_I | BL liana | 6 | 1 | Ecuador: Azuay, along the road from Cuenca to Molleturo, in forest thickets (3 200 m a.s.l.) | S 2.8289 | W 79.1363 | P. Sklenář & J. Karbulková | 11116 |
| *S. involucratus* | 13_I | BL liana | 5 | 1 | Ecuador: Carchi, Volcan Chiles, margin of the road Tufino - Maldonado, ca 1.5 km ESE of Laguna Verde (3 910 m a.s.l.) | N 0.7994 | W 77.9153 | P. Sklenář, E. Rejzková, F. Kolář | 11504 |
| *S. involucratus* | 14_I | BL liana | 3 | 2 | Colombia: Caldas-Tolima, Los Nevados, paramo las Letras, along the road from Honda to Manizales, ca 2 km NE of Letras (3660 m a.s.l.) | N 5.038 | W 75.3342 | Fabio, P. Sklenář et al. | FAA 628 |
| *S. involucratus* | 15_I | BL liana | 5 | –– | Ecuador: Sucumbios, Páramo El Mirador, ca 6 km to the east of Huaca (3700 m a.s.l.) | N 0.6163 | W 77.6717 | P. Sklenář, E. Rejzková, F. Kolář | 11533 |
| *S. involucratus* | 16_I | BL liana | 1 | –– | Ecuador: Imbabura, Volcan Cotacachi, left of the trail from the TV antennas towards the summit, near rocky outcrops (4 190 m a.s.l.) | N 0.3455 | W 78.3442 | P. Sklenář, E. Rejzková, F. Kolář | 11539 |
| *S. involucratus* | 17_I | BL liana | 5 | 1 | Colombia: Cauca, Paramo de Purace, along the footpath from Pilimbala to Volcan Purace (3 817 m a.s.l.) | N 2.3474 | W 76.3993 | E. Rejzková, F. Kolář & D. Vasquez | 39 |
| *S. involucratus* | 18_I | BL liana | 5 | –– | Ecuador: Pichincha - Cotopaxi, Páramo de Iliniza, along the trail from the parking place towads the Ilinizas (4 290 m a.s.l.) | S 0.6428 | W 78.6963 | P. Sklenář, E. Rejzková, F. Kolář | 11555 |
| *S. involucratus* | 19_I | BL liana | 5 | 1 | Ecuador: Cotopaxi, Páramo de Quispicacha, on both sides of the ridge of Puncungusacha, ca 10 km to the west of Quindigua (4 380 m a.s.l.) | S 1.0786 | W 78.8317 | P. Sklenář, E. Rejzková, F. Kolář | 11566 |
| *S. involucratus* | 20_I | BL liana | 2 | 1 | Ecuador: Carchi, road from Laguna Voladero towards Tulcan (3 700 m a.s.l.) | S 0.6833 | W 77.8833 | P. Sklenář & J. Karbulková | 11114 |
| *S. involucratus* | 21_I | BL liana | 5 | 1 | Ecuador: Napo, Road San Miguel de Salcedo-Tena (3 490 m a.s.l.) | S 0.9834 | W 78.3397 | P. Sklenář & A. Kučerová | 11031 |
| *S. involucratus* | 22_I | BL liana | 6 | 1 | Ecuacor: Pichincha, Along the trail from the antennas towards the summit of Atacazo (4 190 m a.s.l.) | S 0.3513 | W 78.6178 | P. Sklenář, A. Kučerová & P. Macek | 11067 |
| *S. involucratus* | 23_I | BL liana | 2 | –– | Ecuador: Chimborazo, Volcan Altar, around Cerro Quilimas, margin of the road from Alao to the valley of Rio Alao, ca 6 km NE of Alao (3 260 m a.s.l.) | S 1.8848 | W 78.4688 | P Sklenář & E Rejzková | 11595 |
| *S. involucratus* | 24_I | BL liana | 4 | –– | Ecuador: Loja, Paramo de Fierro Urco, to the southwest of Saraguro (3 680 m a.s.l.) | S 3.695 | W 79.3486 | P. Sklenář, J. Macková & P. Macek | 12015 |
| *S. involucratus* | 25_I | BL liana | 4 | –– | Colombia: Cauca, Paramo de Moras road from Silvia to Mosoco, ridges between the road and Cerro de Penas Blancas (3 500 m a.s.l.) | N 2.71 | W 76.2167 | P. Sklenář | 12266 |
| *S. involucratus* | 26_I | BL liana | 2 | –– | Colombia: Valle de Cauca, Paramo de Tinajas, Cordillera Central, road from Florida towards the mountain pass (3 790 m a.s.l.) | N 3.3393 | W 76.0636 | P. Sklenář & D. Vasquez | 12271 |
| *S. involucratus* | 27_I | BL liana | 5 | –– | Ecuador: Napo, Paramo de Antisana, NE side of the mountain (4 150 m a.s.l.) | S 0.4517 | W 78.1256 | P. Sklenář | 10014 |
| *S. josei* | 28_J | NL ascending subshrub | 6 | 1 | Ecuador: Loja, Cordillera las Lagunillas (de Sabanilla), Páramo de las Lagunas Negras (3 330 m a.s.l.) | S 4.7106 | W 79.4367 | P. Sklenář, J. Macková & P. Macek | 12027 |
| *S. lingulatus* | 29_L | NL ascending subshrub | 2 | –– | Ecuador: Pichincha, grass páramo on the southern side of Cayambe, along the road towards the refugio (3 900 m a.s.l.) | S 0.0242 | W 78.0519 | P. Sklenář & A. Kučerová | 11080 |
| *S. lingulatus* | 30_L | NL ascending subshrub | 6 | 1 | Ecuador: Azuay, superpáramo vegetation to the N from the pass of the road Cuenca-Molleturo (4 300 m a.s.l.) | S 2.7696 | W 79.2433 | P. Sklenář & J. Karbulková | 11121 |
| *S. lingulatus* | 31_L | NL ascending subshrub | 6 | 1 | Ecuador: Morona Santiago, Mountain pass of the road Gualaceo-Limon, along the way from the pass towards the antennas (3 470 m a.s.l.) | S 3.0033 | W 78.6614 | P. Sklenář & J. Karbulková | 11162 |
| *S. lingulatus* | 32_L | NL ascending subshrub | 3 | 1 | Ecuador: Imbabura, Volcan Cotacachi, margin of the 4 WD road from Laguna Cuicocha north to the TV antennas, near the antennas (4 010 m a.s.l.) | N 0.3323 | W 78.3389 | P. Sklenář, E. Rejzková, F. Kolář | 11538 |
| *S. lingulatus* | 33_L | NL ascending subshrub | 5 | –– | Ecuador: Chimborazo, páramo to the south of Chimborazo (4 270 m a.s.l.) | S 1.5355 | W 78.8809 | P. Sklenář & A. Kučerová | 11035 |
| *S. lingulatus* | 34_L | NL ascending subshrub | 5 | 1 | Ecuador: Chimborazo, Páramo Chanlor, to the west of Guamote (4 030 m a.s.l.) | S 1.9534 | W 78.7933 | P. Sklenář & A. Kučerová | 11038 |
| *S. lingulatus* | 35_L | NL ascending subshrub | 4 | 2 | Ecuador: Napo, páramo on the western side of Antisana (4 500 m a.s.l.) | S 0.4667 | W 78.1667 | P. Sklenář | 11077 |
| *S. lingulatus* | 36_L | NL ascending subshrub | 4 | 1 | Ecuador: Pichincha, around the upper antennas of Atacazo (4 160 m a.s.l.) | S 0.3464 | W 78.6158 | P. Sklenář, A. Kučerová & P. Macek | 11074 |
| *S. lingulatus* | 37_L | NL ascending subshrub | 5 | –– | Ecuador: Cotopaxi, páramo de Quispicacha, ca 0.5 km below the pass to Quebrada Tauricucho (4 150 m a.s.l.) | S 1.072 | W 78.8573 | P. Sklenář, E. Rejzková, F. Kolář | 11569 |
| *S. longepenicillatus* | 38_Lo | NL ascending subshrub | 4 | 1 | Venezuela: Mérida, páramo de Mucubají, trail from Laguna de Mucubají to the Cascadas and Laguna Negra (3 640 m a.s.l.) | N 8.7802 | W 70.8212 | P. Sklenář, P. Ubiergo et al. | 10205 |
| *S. longepenicillatus* | 39_Lo | NL ascending subshrub | 2 | –– | Venezuela: Táchira: páramo Colorado, near the road from Queniquea to El Cobre (3 080 m a.s.l.) | N 7.9406 | W 72.0794 | P. Sklenář, P. Ubiergo et al. | 10377 |
| *S. longepenicillatus* | 40_Lo | NL ascending subshrub | 1 | –– | Venezuela: Táchira: northern reaches of the Páramo del Batallon, to the east from La Grita (3 310 m a.s.l.) | N 8.1619 | W 71.8989 | P. Sklenář, P. Ubiergo et al. | 10417 |
| *S. longepenicillatus* | 41_Lo | NL ascending subshrub | 3 | 1 | Venezuela: Mérida, Páramo de Aguila, along the road from Mucuchies towards the pass de Aguila, northern side of Cerro El Balcón (3 980 m a.s.l.) | N 8.8364 | W 70.8321 | P. Sklenář, P. Ubiergo et al. | 10120 |
| *S. longepenicillatus* | 42_Lo | NL ascending subshrub | 5 | 1 | Venezuela: Mérida/Trujillo, Alto del Arenal, páramo south-east of Tuname along the road to Santo Domingo (3 730 m a.s.l.) | N 9.0294 | W 70.5844 | P. Sklenář, P. Ubiergo et al. | 10279 |
| *S. longepenicillatus* |  | NL ascending subshrub | –– | 1 | Venezuela: Mérida, Páramo de Mucuchíes, road pass Aguila to Pinango, near the antennas (4240 m a.s.l.) | N8.8584 | W70.8261 | P. Sklenář, P. Ubiergo et al. | 10122 |
| *S. mojandensis* | 43_Mo | BL rosette herb | 2 | 1 | Ecuador: Carchi, Volcan Chiles, margin of the road Tufino - Maldonado, ca 1.5 km ESE of Laguna Verde (3 910 m a.s.l.) | N 0.7994 | W 77.9153 | P. Sklenář, E. Rejzková, F. Kolář | 11507 |
| *S. mojandensis* | 44_Mo | BL rosette herb | 1 | 1 | Ecuador: Cotopaxi, Páramo de Lagunas de Anteojos, to the south of Latacunga (3 930 m a.s.l.) | S 0.9781 | W 78.3893 | P. Sklenář & A. Kučerová | 11028 |
| *S. mojandensis* | 45_Mo | BL rosette herb | 1 | 1 | Colombia: Valle de Cauca, Paramo de Tinajas, road from Florida towards the mountain pass (3 790 m a.s.l.) | N 3.3393 | W 76.0636 | P. Sklenář & D. Vasquez | 12272 |
| *S. otophorus* | 46_Ot | NL liana | 4 | –– | Ecuador: Pichincha, northern side of Nevado Cayambe, along the road from Laguna San Marcos towards antennas (3750 m a.s.l.) | N 0.1011 | W 77.9772 | P. Sklenář & E. Rejzková | 10713 |
| *S. otophorus* | 47_Ot | NL liana | 6 | 1 | Ecuador: Chimborazo, Volcan Altar, around Cerro Quilimas, along the trail Alao-Huamboya, ca 1 km N of the bridge across Rio Alao (3 630 m a.s.l.) | S 2.3342 | W 78.4458 | P. Sklenář, E. Rejzková, F. Kolář | 11599 |
| *S. otophorus* | 48_Ot | NL liana | 5 | –– | Ecuador: Loja, Parque Nacional Podocarpus, paramo near the summit of Cerro Toledo (3 400 m a.s.l.) | S 4.3917 | W 79.1125 | P. Sklenář, J. Macková & P. Macek | 12072 |
| *S. otophorus* | 49_Ot | NL liana | 2 | –– | Ecuador: Loja, Paramo de Fierro Urco, to the southwest of Saraguro (3 720 m a.s.l.) | S 3.6908 | W 79.3525 | P. Sklenář, J. Macková & P. Macek | 12007 |
| *S. otophorus* | 50_Ot | NL liana | 5 | 1 | Ecuador: Loja, Cordillera las Lagunillas (de Sabanilla), páramo de las Lagunas Negras (3 400 m a.s.l.) | S 4.7111 | W 79.4308 | P. Sklenář, J. Macková & P. Macek | 12045 |
| *S. otophorus* | 51_Ot | NL ascending subshrub | 4 | 1 | Colombia: Boyacá, Sierra Nevada del Cocuy, valley of the Rio Lagunillas, shrubby vegetation around lagunas (3 940 m a.s.l.) | N 6.3644 | W 72.3331 | P. Sklenář, E. Dušková et al. | 12211 |
| *S. otophorus* | 52_Ot | NL liana | 3 | –– | Colombia: Antioquia, paramo Frontino, trail towards Alto de Burros (3 580 m a.s.l.) | N 6.4475 | W 76.0845 | P. Sklenář, E. Dušková et al. | 12240 |
| *S. otophorus* | 53_Ot | NL liana | 4 | –– | Colombia: Cauca, paramo de Moras road from Silvia to Mosoco, ridges between the road and Cerro de Penas Blancas (3 600 m a.s.l.) | N 2.71 | W 76.2167 | P. Sklenář | 12267 |
| *S. otophorus* | 54_Ot | NL liana | 3 | 1 | Ecuador: Azuay, superparamo vegetation to the N from the pass of the road Cuenca-Molleturo, mountain ridge towards Cerro Amarillo (4 300 m a.s.l.) | S 2.7696 | W 79.2433 | P. Sklenář & J. Karbulková | 11117 |
| *S. otophorus* | 55_Ot | NL liana | 2 | –– | Colombia: Valle de Cauca, Paramo de Tinajas, grass paramo above the Laguna Guayabal (3 790 m a.s.l.) | N 3.3393 | W 76.0636 | P. Sklenář & D. Vasquez | 12276 |
| *S. otophorus* | 56_Ot | NL liana | 5 | 1 | Colombia: Cauca, paramo del Letrero, trail from Valencia towards Laguna Santiago and Laguna Suramerica (3 700 m a.s.l.) | N 1.9206 | W 76.5956 | P. Sklenář, E. Dušková et al. | 12337 |
| *S. otophorus* | 57_Ot | BL liana | 5 | –– | Colombia: Quindio-Tolima, Cerro Campanario, on the paved road leading to the military anntenas at the mountain ridge (3 590 m a.s.l.) | N 4.4503 | W 75.5773 | P. Sklenář, E. Dušková et al. | 12343 |
| *S. otophorus* | 58_Ot | BL liana | 4 | 1 | Colombia: Cundinamarca, paramo de Chingaza, trail from Laguna Chingaza to Laguna de Media (3500 m a.s.l.) | N 4.5098 | W 73.743 | P. Sklenář & F. Kolář | 12349 |
| *S. otophorus* | 59_Ot | BL liana | 5 | 1 | Colombia: Boyacá, paramo de Pisba, shrubby vegetation on slopes to the west of Rio Arzobispo (3 420 m a.s.l.) | N 5.9503 | W 72.5915 | P. Sklenář & E. Dušková | 12373 |
| *S. otophorus* | 60_Ot | NL liana | 6 | 1 | Colombia: Narińo, Volcan Galeras, road from Pasto to the crater (3 520 m a.s.l.) | N 1.2292 | W 77.3392 | P. Sklenář & E. Dušková | 12382 |
| *S. otophorus* | 61_Ot | NL liana | 2 | –– | Colombia: Nariňo, Volcan Azufral, along the path from Tuquerres to Laguna Verde, ca 1 km ENE of the laguna (3 890 m a.s.l.) | N 1.092 | W 77.7085 | F. Kolář | 53 |
| *S. otophorus* | 62_Ot | NL liana | 4 | –– | Ecuador: Morona Santiago, along the road Gualaceo-Limon, humid bamboo subparamo with scattered shrubs (3 320 m a.s.l.) | S 3.0005 | W 78.665 | P. Sklenář & J. Karbulková | 11163 |
| *S. otophorus* | 63_Ot | NL liana | 6 | 1 | Ecuador: Carchi, Volcan Chiles, margin of the road Tufino - Maldonado, ca 1.5 km ESE of Laguna Verde (3 910 m a.s.l.) | N 0.7994 | W 77.9153 | P. Sklenář, E. Rejzková, F. Kolář | 11508 |
| *S. otophorus* | 64_Ot | NL liana | 5 | –– | Colombia: Caldas, Los Nevados, shrubs along the road from Termales to Nevado El Ruiz (3 860 m a.s.l.) | N 4.9582 | W 75.3579 | Fabio, P. Sklenář et al. | FAA 631 |
| *S. superandinus* | 65_O | NL erect subshrub | 1 | –– | Ecuador: Cotopaxi, paramo to the west of Quilindana (4 120 m a.s.l.) | S 0.8008 | W 78.3884 | P. Sklenář | 10045 |
| *S. superandinus* | 66_O | NL erect subshrub | 4 | 1 | Ecuador: Imbabura, páramo de Mojanda, on the SW slope of the peak Nudo de Mojanda (4 130 m a.s.l.) | N 0.1186 | W 78.26 | P. Sklenář & E. Rejzková | 10744 |
| *S. superandinus* | 67_O | NL erect subshrub | 5 | –– | Ecuador: Pichincha, páramo on the southern side of Cayambe, along the road towards the refugio, bellow the steep rock walls (4 430 m a.s.l.) | S 0.0246 | W 78.0501 | P. Sklenář & A. Kučerová | 11092 |
| *S. superandinus* | 68_O | NL erect subshrub | 6 | 1 | Ecuador: Azuay, superparamo vegetation to the N from the pass of the road Cuenca-Molleturo, mountain ridge towards Cerro Amarillo (4 300 m a.s.l.) | S 2.7696 | W 79.2433 | P. Sklenář & J. Karbulková | 11118 |
| *S. superandinus* | 69_O | NL erect subshrub | 2 | –– | Ecuador: Carchi, Volcan Chiles, ca 0.5 km N of the antennas, ca 1 km N of the pass with the road Tufino - Maldonado (4 160 m a.s.l.) | N 0.8063 | W 77.9422 | P. Sklenář, E. Rejzková, F. Kolář | 11518 |
| *S. superandinus* | 70_O | NL erect subshrub | 1 | 1 | Ecuador: Imbabura, Volcan Cotacachi, along the trail from the TV antennas towards the summit (4 130 m a.s.l.) | N 0.3437 | W 78.3431 | P. Sklenář, E. Rejzková, F. Kolář | 11540 |
| *S. superandinus* | 71_O | NL erect subshrub | 5 | 1 | Ecuador: Pichincha, along the trail from the antennas towards the summit of Atacazo (4160 m a.s.l.) | S 0.3513 | W 78.6178 | P. Sklenář, A. Kučerová & P. Macek | 11071 |
| *S. superandinus* | 72_O | NL erect subshrub | 4 | 1 | Ecuador: Cotopaxi, Páramo de Lagunas de Anteojos, to the south of Latacunga (3 930 m a.s.l.) | S 1.5355 | W 78.8809 | P. Sklenář & A. Kučerová | 11032 |
| *S. superandinus* | 73_O | NL erect subshrub | 2 | 1 | Ecuador: Chimborazo, páramo de Osogochi, grass páramo on the slopes above Laguna Cubillín (3 970 m a.s.l.) | S 2.287 | W 78.5863 | P. Sklenář & J. Karbulková | 11188 |
| *S. superandinus* | 74_O | NL erect subshrub | 5 | –– | Ecuador: Imbabura, páramo de Quispicacha, pass between valley of Rio Pigua and Quebrada Tauricucho, ca 5 km ESE of Chinipamba (4 130 m a.s.l.) | S 1.0788 | W 78.8474 | P. Sklenář, E. Rejzková, F. Kolář | 11568 |
| *S. superandinus* | 75_O | NL erect subshrub | 6 | –– | Colombia: Valle de Cauca, paramo de Tinajas, Cordillera Central, rocky summit of the mountain ridge above Laguna Guayabal (4 160 m a.s.l.) | N 3.3445 | W 76.0541 | P. Sklenář & D. Vasquez | 12288 |
| *S. superandinus* | 76_O | NL erect subshrub | 1 | –– | Colombia: Cauca, Volcan Purace, the trail from the sulphur mine to the northern side of the crater (4 160 m a.s.l.) | N 2.34 | W 76.4037 | P. Sklenář, E. Dušková et al. | 12316 |
| *S. superandinus* |  | NL erect subshrub | –– | 1 | Ecuador: Pichincha, Páramo de Iliniza, along the trail from the parking place towads the Ilinizas (4294 m a.s.l.) | S0.6428 | W78.6963 | P. Sklenář, E. Rejzková, F. Kolář | 11559 |
| *S. patens* | 77_P | BL liana | 1 | –– | Ecuador: Carchi - Sucumbios, Páramo El Mirador, ca 6 km to the east of Huaca (3 230 m a.s.l.) | N 0.647 | W 77.674 | P. Sklenář, E. Rejzková, F. Kolář | 11530 |
| *S. patens* | 78_P | BL liana | 2 | –– | Ecuador: Imbabura, Volcan Cotacachi, margin of the 4 WD road from Laguna Cuicocha north to the TV antennas (3 390 m a.s.l.) | N 0.313 | W 78.3526 | P. Sklenář, E. Rejzková, F. Kolář | 11536 |
| *S. patens* | 79_P | BL liana | 2 | 1 | Ecuador: Cotopaxi, páramo Quispicacha (3 730 m a.s.l.) | S 1.0453 | W 78.9673 | P. Sklenář, E. Rejzková, F. Kolář | 11565 |
| *S. patens* | 80_P | BL liana | 3 | 1 | Ecuador: Carchi, road from El Angel towards Laguna Voladero (3 260 m a.s.l.) | N 0.8283 | W 77.9001 | P. Sklenář & J. Karbulková | 11113 |
| *S. patens* | 81_P | BL liana | 3 | –– | Colombia: Cauca, Purace, margin of the road Popayan-La Plata, 3 km ESE of municipio Purace (3 290 m a.s.l.) | N 2.374 | W 76.4061 | E. Rejzková, F. Kolář, D. Vasquez | 35 |
| *S. patens* | 82_P | BL liana | 1 | –– | Ecuador: Pichincha, northern side of Pichincha, along the road to the forest reserve Jocotoco (3 650 m a.s.l.) | S 0.115 | W 78.5742 | P. Sklenář & A. Soukup | 12097 |
| *S. patens* | 83_P | BL liana | 6 | –– | Colombia: Cauca, paramo del Letrero, trail from Valencia towards Laguna Santiago and Laguna Suramerica (3 230 m a.s.l.) | N 1.9157 | W 76.6228 | P. Sklenář, E. Dušková et al. | 12333 |
| *S. patens* | 84_P | BL liana | 2 | –– | Colombia: Narińo, Volcan Galeras, road from Pasto to the crater (3 490 m a.s.l.) | N 1.2265 | W 77.3374 | P. Sklenář & E. Dušková | 12377 |
| *S. patens* | 85_P | BL liana | 2 | –– | Colombia: Narińo, Volcan Azufral, margin of the road from Tuquerres to Laguna Verde, ca 6 km W of Tuquerres (3 500 m a.s.l.) | N 1.0925 | W 77.6698 | F. Kolář | 51 |
| *S. patens* | 86_P | BL liana | 1 | 1 | Ecuador: Carchi, Volcan Chiles, margin of the road Tufino - Maldonado, ca 1.5 km ESE of Laguna Verde (3 910 m a.s.l.) | N 0.7889 | W 77.8798 | P. Sklenář, E. Rejzková, F. Kolář | 11501 |
| *S. aff. quitensis* | 87_Pi | NL/BL subshrub | 2 | –– | Colombia: Valle de Cauca, paramo de Tinajas, Cordillera Central, rocky summit of the mountain ridge above Laguna Guayabal (4 160 m a.s.l.) | N 3.3445 | W 76.0541 | P. Sklenář & D. Vasquez | 12289 |
| *S. aff. quitensis* | 88_Pi | NL/BL subshrub | 4 | 1 | Ecuador: Carchi, Volcan Chiles, margin of the road Tufino - Maldonado, ca 1.5 km ESE of Laguna Verde (3 910 m a.s.l.) | N 0.7994 | W 77.9153 | P. Sklenář, E. Rejzková, F. Kolář | 11509 |
| *S. aff. quitensis* | 89_Pi | NL/BL subshrub | 2 | –– | Ecuador: Imbabura, Volcan Cotacachi, southern slopes of the mountain (4 370 m a.s.l.) | N 0.3536 | W 78.3494 | P. Sklenář, E. Rejzková, F. Kolář | 11546 |
| *S. aff. quitensis* | 90_Pi | NL/BL subshrub | 2 | –– | Ecuador: Pichincha, around the upper antennas of Atacazo, lower superpáramo vegetation (4 160 m a.s.l.) | S 0.3464 | W 78.6158 | P. Sklenář, A. Kučerová & P. Macek | 11075 |
| *S. aff. quitensis* | 91_Pi | NL/BL subshrub | 1 | –– | Ecuador: Pichincha, páramo on the southern side of Cayambe, along the road towards the refugio, bellow the steep rock walls (4 250 m a.s.l.) | S 0.0167 | W 78.05 | P. Sklenář & A. Kučerová | 11084 |
| *S. aff. quitensis* | 92_Pi | NL/BL subshrub | 1 | –– | Ecuador: Pichincha, páramo on the southern side of Cayambe, along the road towards the refugio, bellow the steep rock walls (4 430 m a.s.l.) | S 0.0246 | W 78.0501 | P. Sklenář & A. Kučerová | 11091 |
| *S. aff. quitensis* | 93_Pi | NL/BL subshrub | 2 | –– | Ecuador: Pichincha, Rucu Pichincha, along the trail from the Teleferico to the summit (4 550 m a.s.l.) | S 0.1611 | W 78.5653 | P. Sklenář & J. Karbulková | 11191 |
| *S. puracensis* | 94_Pu | NL ascending subshrub | 5 | 2 | Colombia: Cauca, Volcan Purace, the trail from the sulphur mine to the northern side of the crater (4 110 m a.s.l.) | N 2.3324 | W 76.3943 | P. Sklenář, E. Dušková et al. | 12311 |
| *S. imbaburensis* | 95_S | NL ascending subshrub | 3 | –– | Ecuador: Imbabura, paramo de Mojanda, on the SW slope of the peak Nudo de Mojanda (4 020 m a.s.l.) | N 0.1147 | W 78.2633 | P. Sklenář & E. Rejzková | 10752 |
| *S. imbaburensis* | 96_S | NL ascending subshrub | 5 | 1 | Ecuador: Pichincha, páramo on the southern side of Cayambe, along the road towards the refugio, bellow the steep rock walls (4 430 m a.s.l.) | S 0.0246 | W 78.0501 | P. Sklenář & A. Kučerová | 11093 |
| *S. imbaburensis* | 97_S | NL ascending subshrub | 2 | 1 | Ecuador: Imbabura, Volcan Cotacachi, margin of the 4 WD road from Laguna Cuicocha north to the TV antennas, near the antennas (4010 m a.s.l.) | N 0.3323 | W 78.3389 | P. Sklenář, E. Rejzková, F. Kolář | 11547 |
| *S. subinvolucratus* | 98_SI | BL liana | 4 | –– | Ecuador: Pichincha, páramo on the NW side of Atacazo (4 160 m a.s.l.) | S 0.3867 | W 78.6002 | P. Sklenář, A. Kučerová & P. Macek | 11076 |
| *S. superparamensis* | 99_SP | NL ascending subshrub | 2 | –– | Ecuador: Napo, superparamo vegetation on the western side of Antisana (4 600 m a.s.l.) | S 0.4722 | W 78.1653 | P. Sklenář & D. Carrate | 10739 |
| *S. superparamensis* | 100_SP | NL ascending subshrub | 3 | 1 | Ecuador: Carchi, Volcan Chiles, margin of the road Tufino - Maldonado, around Laguna Verde (4 030 m a.s.l.) | N 0.8006 | W 77.9358 | P. Sklenář, E. Rejzková, F. Kolář | 11510 |
| *S. superparamensis* | 101_SP | NL ascending subshrub | 4 | 1 | Ecuador: Imbabura, Volcan Cotacachi, southern slopes of the mountain (4 370 m a.s.l.) | N 0.3536 | W 78.3494 | P. Sklenář, E. Rejzková, F. Kolář | 11544 |
| *S. superparamensis* | 102_SP | NL ascending subshrub | 5 | 2 | Ecuador: Napo, Páramo of Antisana, NE side of the mountain (4 400 m a.s.l.) | S 0.45 | W 78.1333 | P. Sklenář | 11079 |
| *S. iscoensis* | 103_3 | BL (sub) shrub | 2 | –– | Ecuador: Cotopaxi, Loma Ingapirca, right turn-of from the road Zumbagua - Angamarca, ca 5 km NNW of Quindigua (3 730 m a.s.l.) | S 1.0453 | W 78.9673 | P. Sklenář, E. Rejzková, F. Kolář | 11567 |
| *S. iscoensis* | 104_3 | BL (sub)shrub | 1 | –– | Ecuador: Chimborazo, Candelaria, road from the village towards lagunas (3 240 m a.s.l.) | S 1.6486 | W 78.5058 | P. Sklenář, J. Macková & P. Macek | 12094 |
| *S. nivalis* | 105_CN | NL herb to subshrub | 4 | 1 | Ecuador: Carchi, Volcan Chiles, ca 0.5 km N of the antennas, ca 1 km N of the pass with the road Tufino - Maldonado (4 030 m a.s.l.) | N 0.8006 | W 77.9422 | P. Sklenář, E. Rejzková, F. Kolář | 11515 |
| *S. nivalis* | 106_CN | NL herb to subshrub | 4 | 1 | Ecuador: Imbabura, Volcan Cotacachi, southern slopes of the mountain (4 370 m a.s.l.) | N 0.3536 | W 78.3494 | P. Sklenář, E. Rejzková, F. Kolář | 11543 |
| *S. nivalis* | 107_CN | NL herb to subshrub | 5 | 1 | Ecuador: Napo, Volcan Antisana, N side of the mountain, towards the Quebrada of Río Blanco (4 350 m a.s.l.) | S 0.4501 | W 78.1364 | P. Sklenář & F. Kolář | 11580 |
| *S. nivalis* | 108_CN | NL herb to subshrub | 5 | 1 | Ecuador: Tungurahua - Chimborazo - Bolivar, páramo to the SW of Chimborazo, ca 2 km of the road Cruz del Arenal-San Juan (4 270 m a.s.l.) | S 1.5353 | W 78.8808 | P. Sklenář, E. Rejzková, F. Kolář | 11586 |
| *S. alatopetiolatus* |  | BL liana | –– | 1 | Ecuador: Tungurahua, Parque Nacional Llanganatis, near laguna at the western side of Cerro Hermoso (3870 m a.s.l.) | S1.2308 | W78.3012 | P. Sklenář | 13100 |
| *S. canescens* |  | BL rosette herb | –– | 1 | Colombia: Boyaca, Paramo around the Laguna Alcohol (3900 m a.s.l.) | S1.1354 | W78.3694 | P. Sklenář, E. Dušková | 12356 |
| *S. chionogeton* |  | NL rosette herb | –– | 1 | Ecudor: Tungurahua, Parque Nacional Llanganatis, grass paramo above the Laguna Pisayambo (3910 m a.s.l.) | S1.1354 | W78.3694 | P. Sklenář | 12414 |
| *S. cocuyensis* |  | BL rosette herb | –– | 1 | Colombia: Boyaca, Sierra Nevada del Cocuy, valley of the Rio Lagunillas, shrubby vegetation around lagunas (3940 m a.s.l) | N6.0814 | W72.937 | P. Sklenář, E. Dušková, F. Kolář, D. Vásquez | 12212 |
| *S. decipiens* |  | BL liana | –– | 1 | Ecuador: Loja/Zamora-Chinchipe, Road from Jimbura to Las Cienegas (2550 m a.s.l.) | S4.6786 | W79.4517 | P. Sklenář, J. Macková, P. Macek | 12024 |
| *S. decipiens* |  | BL liana | –– | 1 | Ecuador: Loja, Via antigua from Loja to Catamayo, western outskirts of Loja (2380 m a.s.l.) | S4.0164 | W79.2464 | P. Sklenář, J. Macková, P. Macek | 12065 |
| *S. rhizocephalus* |  | Acaulescent herb | –– | 1 | Ecudor: Tungurahua, Parque Nacional Llanganatis, grass paramo above the Laguna Pisayambo (3910 m a.s.l.) | N6.3644 | W72.3331 | P. Sklenář | 12411 |
| *S. doryphyllus* |  | BL liana | –– | 1 | Colombia: Cesar, Cerro del Avion, rocks on the top of the mountain, approx 10 km ESE of Manaure | –– | –– | F. Kolář, D. Vásquez | 12392 |
| *S. doryphyllus* |  | BL liana | –– | 1 | Colombia: Cesar, Páramo de Cerro del Avion, forest close to ruines of the house, SSE of Cerro Pintado, approx 8 km ESE of Manaure | –– | –– | F. Kolář, D. Vásquez | 12393 |
| *S. sp.1* |  | NL herb | –– | 1 | Colombia: Boyaca, Paramo de Pisba, shrubby vegetation on slopes to the west of Rio Arzobispo (3420 m a.s.l) | N5.9503 | W72.5915 | P. Sklenář, E. Dušková | 12376 |
| *S. sp. 2* |  | NL herb |  | 1 | Colombia: Boyaca, Sierra Nevada del Cocuy, valley of the Rio Lagunillas, paramo vegetation along the trail towards the Laguna Cuadrada (4120 m a.s.l) | N6.3536 | W72.3269 | P. Sklenář, E. Dušková, F. Kolář, D. Vásquez | 12210 |
