# Supplementary material for: Growth form evolution and hybridization in Senecio (Asteraceae) from the high equatorial Andes
Source: Ecol Evol. 2017 Jul 10;7(16):6455–68. doi: 10.1002/ece3.3206 (PMC5574811; doi:10.1002/ece3.3206)
Supplement: Supplementary file 2 [file ECE3-7-6455-s002.doc]

*Journal of Biogeography*

**SUPPORTING INFORMATION**

**Article title**: Growth-form evolution and hybridization in *Senecio* (Asteraceae) from the high equatorial Andes

Authors: Dušková et al.

Appendix S2. Supplementary molecular protocols

**DNA extraction**

Lab work was done mostly at the DNA laboratory, Department of Botany, Charles University, Prague, Czech Republic, with some of the extractions done at the Botany and Systematics Lab, Universidad de los Andes, Bogota, Colombia. Total genomic DNA was extracted from silica-dried material using the Invisorb Spin Plant Mini Kit (Invitek) according to the manufacturer’s instructions.

**AFLP protocol**

We used AFLP Core Reagent Kit I (Invitrogen) and AFLP Pre-Amp Primer Mix I (Invitrogen) following manufacturer instructions with several minor modifications, including using five times smaller reaction volumes. Fifteen to 50 ng of genomic DNA was digested for 7.5 hr. at 37°C with 0.5 U of *Eco*RI/*Mse*I (Invitrogen), 1 μl 5× reaction buffer (Invitrogen), and 2.5 μl ddH_2_O. Final incubation for 15 min. at 70°C was done immediately after restriction. Adapters were ligated to the digested fragments by adding 4.8 μl Adapter/Ligation Solution (Invitrogen) and 0.2 U T4 DNA Ligase (Invitrogen) and incubated for 12-16 hr. at 37°C. Pre-amplification reaction was performed with AFLP Pre-Amp Primer Mix I (Invitrogen). For each sample, a pre-amplification mixture containing 0.5 μl DNA from the ligation reaction, 4.0 μl PA mix (Invitrogen), 0.5 μl 10× Buffer for JumpStartRedTaq (Sigma), and 0.1U JumpStartRedTaq DNA Polymerase (Sigma) was placed in a Mastercycler ep S thermal cycler (Eppendorf). Reaction conditions consisted of an initial step of 2 min. at 94°C, then 2 min. at 72°C followed by 20 cycles of 1 sec. at 94°C, 30 sec. at 56°C, and 2 min. at 72°C, with a final extension of 30 min. at 60°C. Once the pre-amplification was complete, selective amplification was performed using 2.5 μl of 5× diluted pre-amplification product as a template, 1 μl 10× Buffer for JumpStartRedTaq (Sigma), 0.2 mM dNTP, 0.5 pmol *Eco*RI-selective fluorescence-labeled primer, 2.5 pmol *Mse*I-selective primer, 0.2 U JumpStartRedTaq DNA Polymerase (Sigma), and 5.1 μl ddH_2_O. Three primer combinations were used for selective amplification: *Eco*RI-ACT (6-FAM labeled) + *Mse*I-CTA, *Eco*RI-ATC (6-FAM labeled) + *Mse*I-CAT, and *Eco*RI-AGG (HEX labeled) + *Mse*I-CAT. For all samples, the reactions were done in a Mastercycler ep S thermal cycler (Eppendorf). Reaction conditions consisted of an initial step of 2 min. at 94°C, then 2 min. at 72°C followed by 8 cycles of 1 sec. at 94°C, 30 sec. at 64°C (reduced by 1°C per cycle), and 2 min. at 72°C, followed by 23 cycles of 1 sec. at 94°C, 30 sec. at 56°C, and 2 min. at 72°C, with a final extension time of 30 min. at 60°C. For each sample, 1 μl of each selective PCR product was combined with 0.25 μl of ROX 500 size standard (AppliedBio). Fragments were resolved on an ABI3130 Avant Genetic Analyzer (AppliedBio).

**ITS sequencing**

PCR amplifications were done in XX μl reaction containing 0.18 mM of each dNTP (Fermentas), 0.23 mM of each primer (Sigma), 0.5 unit of JumpStart REDTaq polymerase (Sigma), 1 × PCR buffer for JumpStart REDTaq (Sigma) and 5 ng of genomic DNA. An initial denaturation step at 94°C for 1 min was followed by 35 cycles of denaturation (94°C for 45 s), annealing (49-52°C for 45 s) and extension (72°C for 1 min) steps, and a final extension at 72°C for 10 min. PCR products were purified using JETQUICK PCR Product Purification Spin Kit (Genomed) and subsequently sequenced (Macrogen, lll).
