# Supplementary material for: Growth form evolution and hybridization in Senecio (Asteraceae) from the high equatorial Andes
Source: Ecol Evol. 2017 Jul 10;7(16):6455–68. doi: 10.1002/ece3.3206 (PMC5574811; doi:10.1002/ece3.3206)
Supplement: Supplementary file 3 [file ECE3-7-6455-s003.doc]

*Journal of Biogeography*

**SUPPORTING INFORMATION**

**Article title**: Growth-form evolution and hybridization in *Senecio* (Asteraceae) from the high equatorial Andes

Authors: Dušková et al.


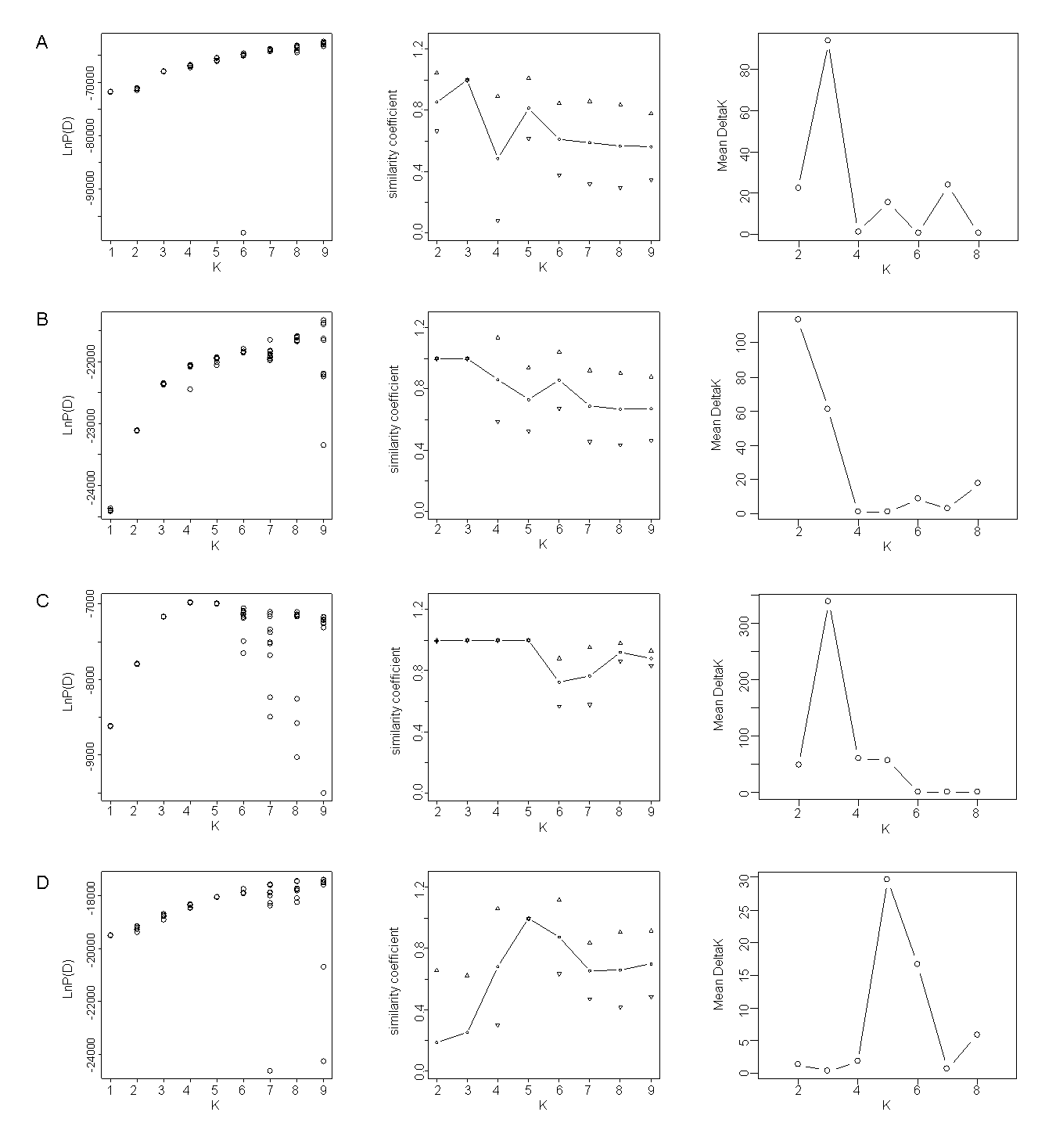
Appendix S3A. Comparison of the results of different Structure runs with increasing K (from K1 to K10, ten replicates each, see Materials and Methods for details). Analysis of entire dataset (A) and separate analyses of individuals assigned only to cluster A (B); cluster B (C); and cluster C (D).


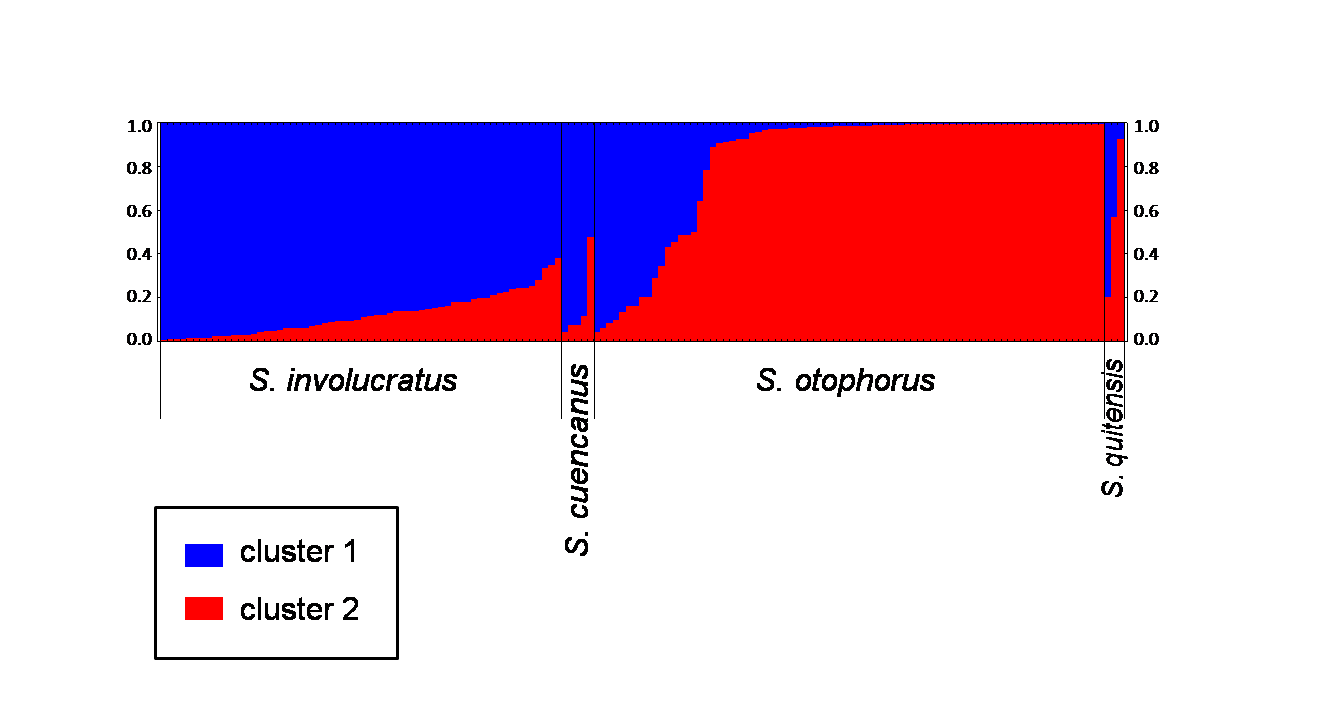


Appendix S3B. Results of Structure analysis of 149 individuals of high-elevation Andean *Senecio* assigned to cluster A for K = 2. Colors indicate posterior probabilities for membership of each individual in the two resulting subgroups.


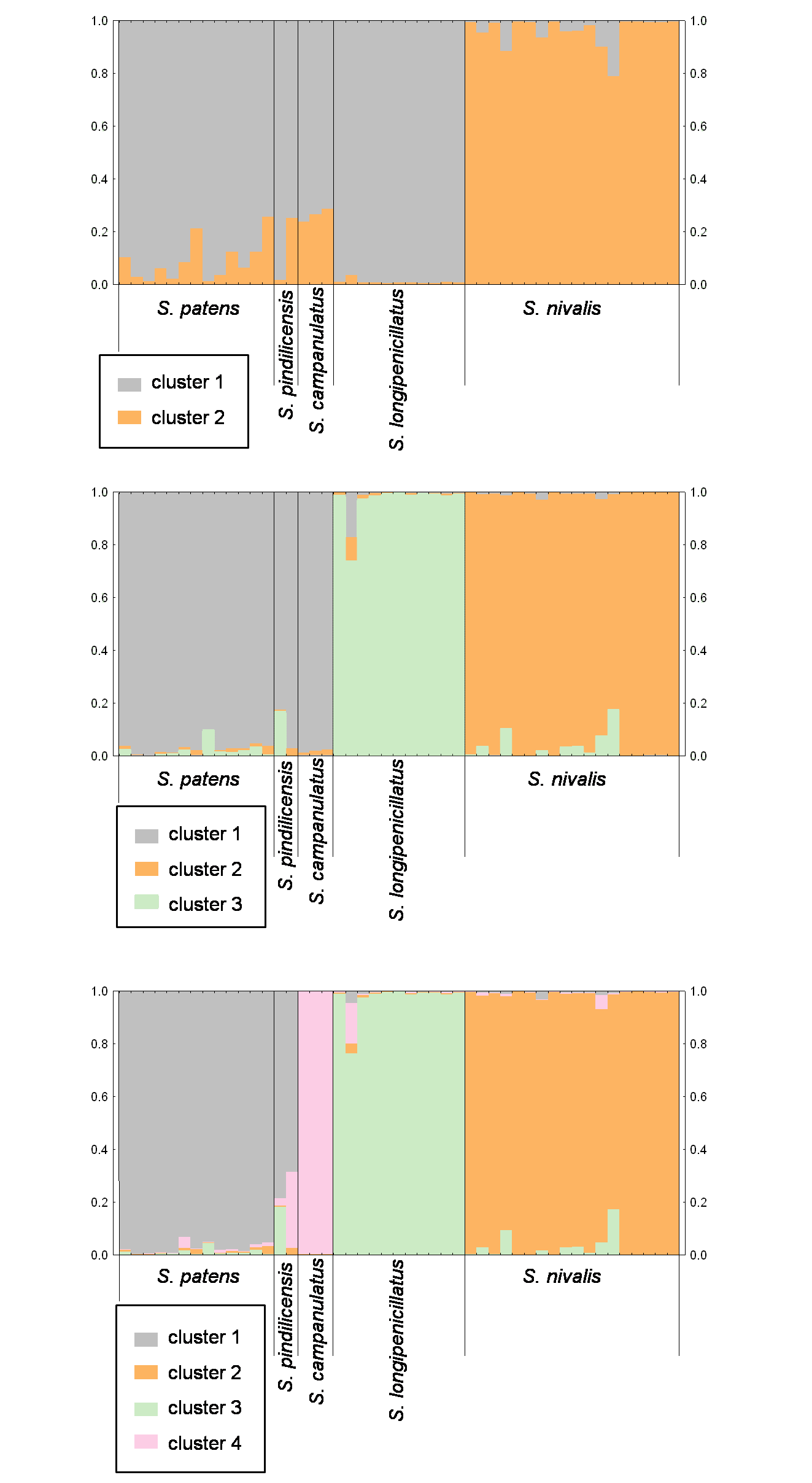


Appendix S3C. Results of Structure analysis of 47 individuals of high-elevation Andean *Senecio* assigned to cluster B for (A) K = 2, (B) K = 3, (C) K = 4. Colors indicate posterior probabilities for membership of each individual in the resulting sub-groups.


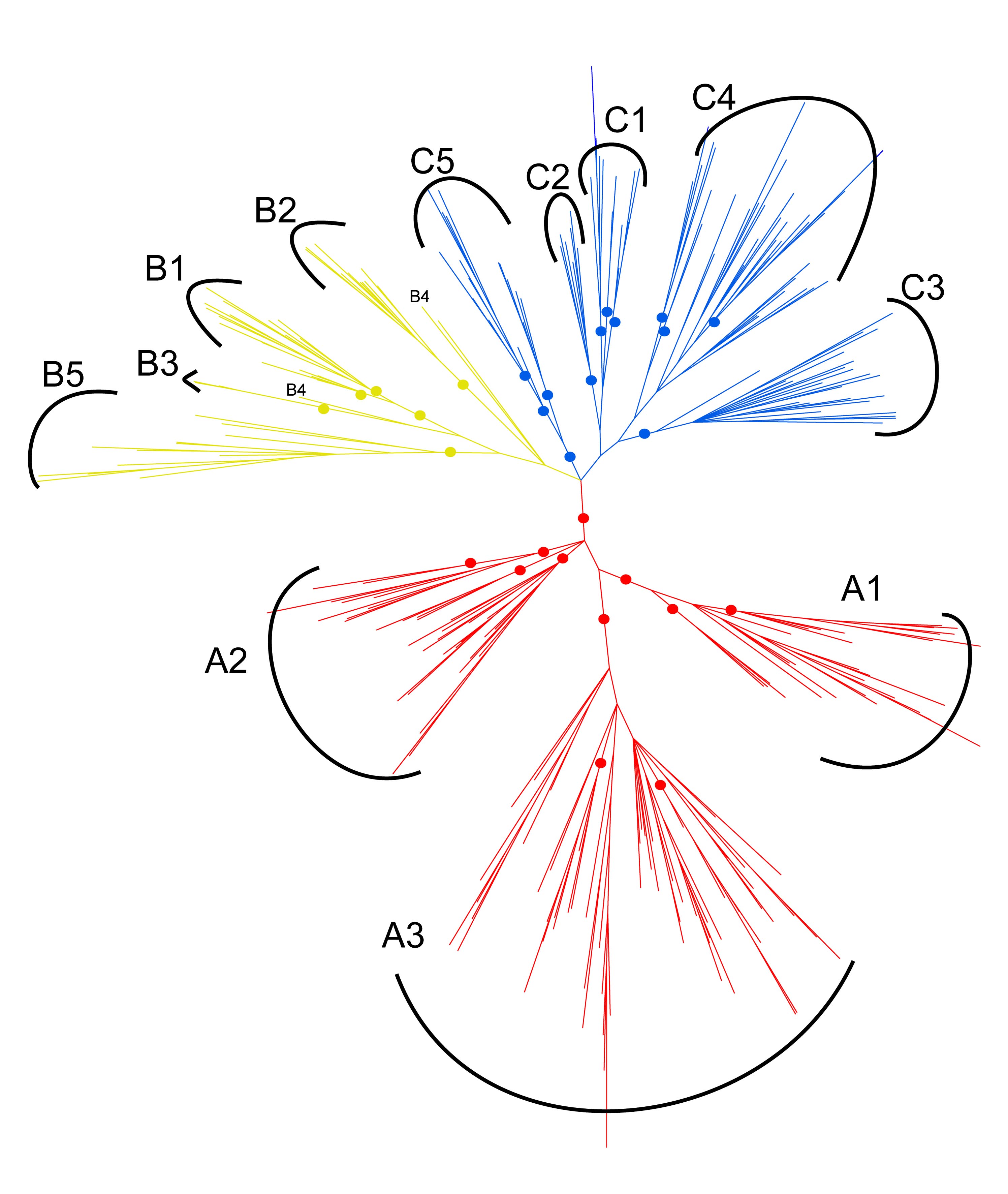


Appendix S3D. Relationships among AFLP phenotypes of 266 non-admixed (see Methods) individuals of former *Lasiocephalus* and *Senecio nivalis* reconstructed in Bayesian framework. Cluster codes correspond with Figs. 3–5; branches with posterior probabilities > 0.95 are marked with dots.


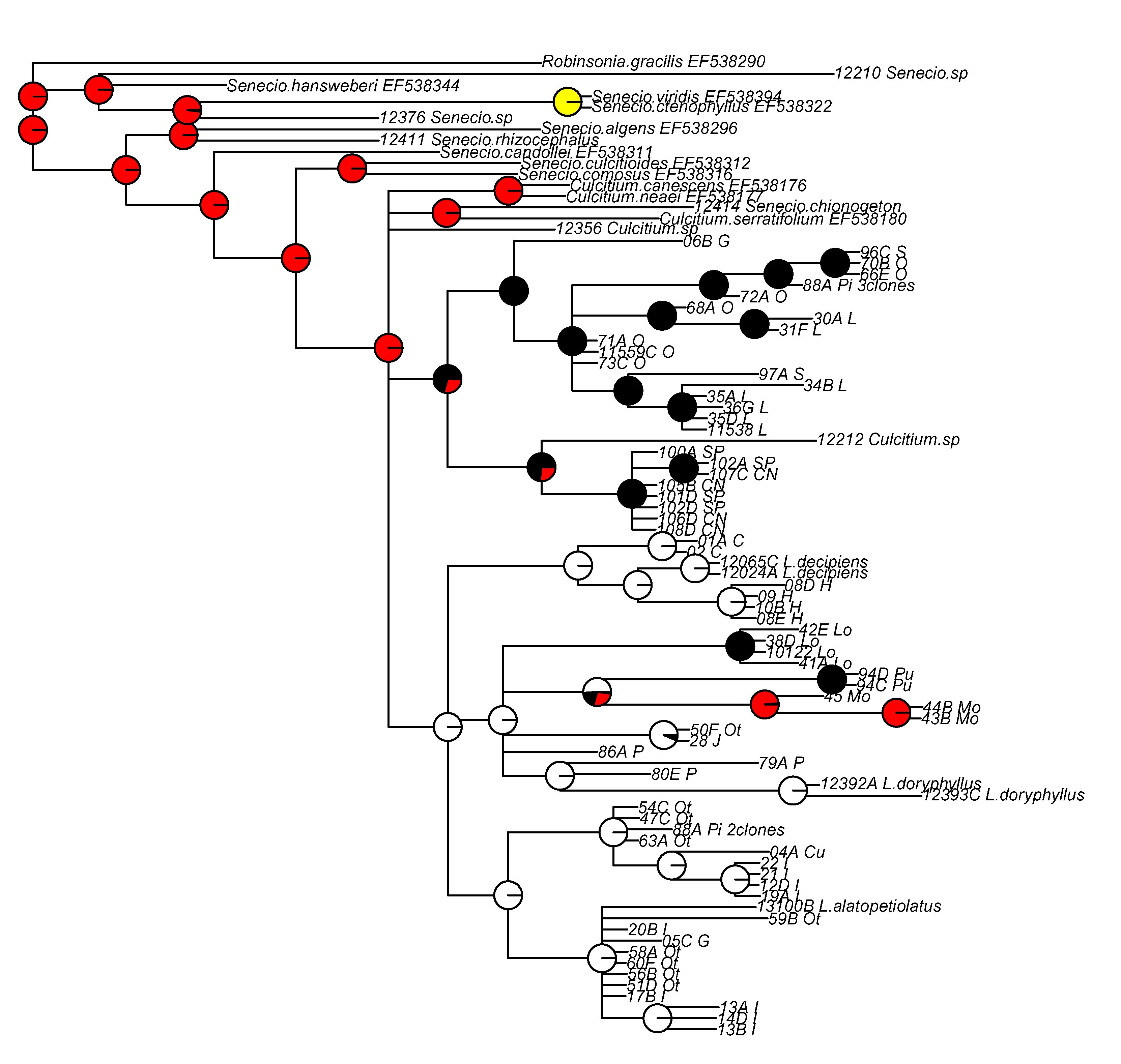


Appendix S3E. Equal rates (ER) model reconstruction of the growth form evolution in the Senecio-*Lasiocephalus*-*Culcitium* species group; basal leaf rosette herb – red, narrow leaved subshrub – black, broad leaved liana – white, shrub – yellow.
